# MicroRNA-200, associated with metastatic breast cancer, promotes traits of mammary luminal progenitor cells

**DOI:** 10.18632/oncotarget.20698

**Published:** 2017-09-07

**Authors:** Lourdes Sánchez-Cid, Mònica Pons, Juan José Lozano, Nuria Rubio, Marta Guerra-Rebollo, Aroa Soriano, Laia Paris-Coderch, Miquel F. Segura, Raquel Fueyo, Judit Arguimbau, Erika Zodda, Raquel Bermudo, Immaculada Alonso, Xavier Caparrós, Marta Cascante, Arash Rafii, Yibin Kang, Marian Martínez-Balbás, Stephen J. Weiss, Jerónimo Blanco, Montserrat Muñoz, Pedro L. Fernández, Timothy M. Thomson

**Affiliations:** ^1^ Institute of Molecular Biology, Spanish National Research Council (IBMB-CSIC), Barcelona, Spain; ^2^ Institut d’Investigacions Biomèdiques August Pi i Sunyer (IDIBAPS), Barcelona, Spain; ^3^ Networked Biomedical Research Center for Hepatic and Digestive Diseases (CIBER-EHD), Instituto de Salud Carlos III, Madrid, Spain; ^4^ Networked Biomedical Research Center for Bioengineering, Biomaterials and Nanomedicine (CIBER-BBN), Instituto de Salud Carlos III, Madrid, Spain; ^5^ Institute of Advanced Chemistry of Catalonia (IQAC-CSIC), Barcelona, Spain; ^6^ Vall d’Hebron Institut de Recerca (VHIR), Universitat Autònoma de Barcelona, Barcelona, Spain; ^7^ Department of Biochemistry and Molecular Biology, Faculty of Biology, Universitat de Barcelona, Barcelona, Spain; ^8^ Department of Obstetrics and Gynecology, Hospital Clinic, Barcelona, Spain; ^9^ Weill Cornell Medical College, Education City, Ar-Rayyan, Qatar; ^10^ Department of Molecular Biology, Princeton University, Princeton, NJ, USA; ^11^ Life Sciences Institute, University of Michigan, Ann Arbor, MI, USA; ^12^ Department of Oncology, Hospital Clínic, Barcelona, Spain; ^13^ Department of Pathology, Hospital Clínic, Barcelona, Spain; ^14^ School of Medicine, University of Barcelona, Barcelona, Spain; ^15^ Current address: Networked Biomedical Research Center for Bioengineering, Biomaterials and Nanomedicine (CIBER-BBN), Instituto de Salud Carlos III, Madrid, Spain

**Keywords:** microRNAs, miR-200, epithelial reprogramming, progenitor luminal cells, invasive ductal breast cancer

## Abstract

MicroRNAs are critical regulators of gene networks in normal and abnormal biological processes. Focusing on invasive ductal breast cancer (IDC), we have found dysregulated expression in tumor samples of several microRNAs, including the miR-200 family, along progression from primary tumors to distant metastases, further reflected in higher blood levels of miR-200b and miR-7 in IDC patients with regional or distant metastases relative to patients with primary node-negative tumors. Forced expression of miR-200s in MCF10CA1h mammary cells induced an enhanced epithelial program, aldehyde dehydrogenase (ALDH) activity, mammosphere growth and ability to form branched tubuloalveolar structures while promoting orthotopic tumor growth and lung colonization *in vivo*. MiR-200s also induced the constitutive activation of the PI3K-Akt signaling through downregulation of PTEN, and the enhanced mammosphere growth and ALDH activity induced in MCF10CA1h cells by miR-200s required the activation of this signaling pathway. Interestingly, the morphology of tumors formed *in vivo* by cells expressing miR-200s was reminiscent of metaplastic breast cancer (MBC). Indeed, the epithelial components of MBC samples expressed significantly higher levels of miR-200s than their mesenchymal components and displayed a marker profile compatible with luminal progenitor cells. We propose that microRNAs of the miR-200 family promote traits of highly proliferative breast luminal progenitor cells, thereby exacerbating the growth and metastatic properties of transformed mammary epithelial cells.

## INTRODUCTION

The acquisition of metastatic properties by cancer cells requires both the occurrence of genetic events that confer a minority of neoplastic cells with inherent self-renewal properties that no longer rely on environmental cues specific to the tissue of origin and also the epigenetic remodeling of selected properties that allow the cells to overcome environmental hurdles to metastasis [1]. As opposed to mutations, epigenetic processes are reversible and are used by cancer cells as a survival strategy that allows them to switch phenotypes in order to optimize adaptation to potentially hostile environmental conditions [1].

MicroRNAs are epigenetic regulators that modulate gene networks that determine the balance between cell invasion *vs*. cohesion, proliferation *vs*. quiescence, and senescence *vs*. cell death [2, 3]. Many microRNAs display dysregulated expression in neoplastic states and either confer or repress the metastatic properties to tumor cells in experimental models [4]. Herein, we have assessed the role of microRNAs in regulating the ability of invasive breast ductal carcinoma (IDC) cells to metastasize to local lymph nodes as a surrogate marker of metastatic potential. We report that the miR-200 family members are significantly upregulated in lymph node (LN) metastases and, by using the MCF10CA1 mammary cell model, that miR-200s induce features of mammary luminal progenitor cells, which may explain the association of enhanced self-renewal, tumorigenesis and metastatic potentials with high levels of expression of miR-200s.

## RESULTS

### Identification of sets of microRNAs dysregulated along IDC metastatic progression

**Figure 1:**
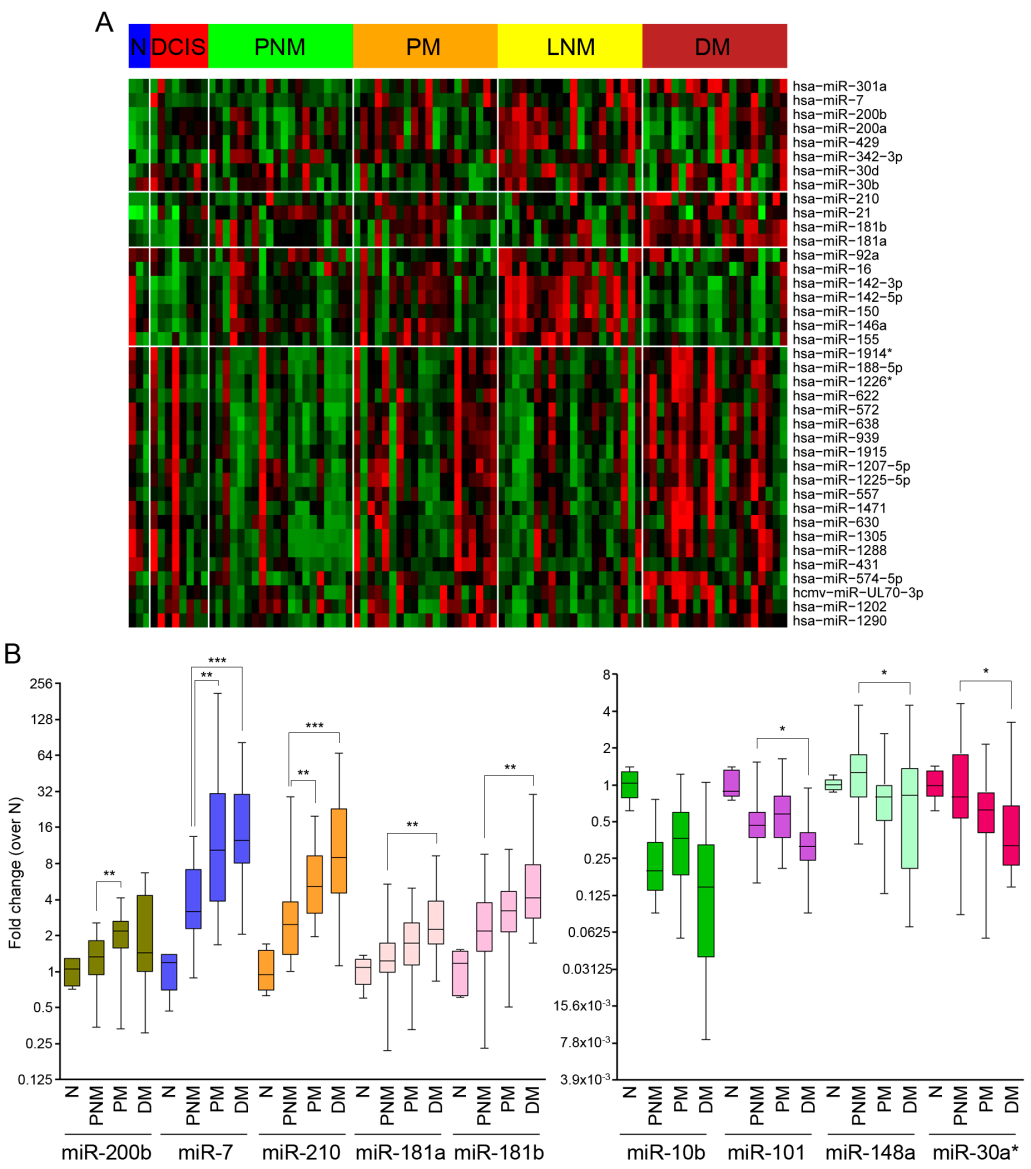
MicroRNAs dysregulated along malignant invasive ductal carcinoma (IDC) progression **A.** Microarray analysis of relative expression levels of miRNAs differentially expressed between samples of normal breast epithelium (N), ductal carcinoma *in situ* (DCIS), primary IDC with no regional lymph node involvement or distant metastasis (PNM), primary IDC with lymph node involvement (PM), matched lymph node metastases (LNM) or distant metastases (DM). The heatmap was generated after probe normalization and selection of differentially expressed miRNAs. **B.** Quantification by qPCR of levels of selected miRNAs along metastatic progression of IDC. Reference probe-normalized values (n^- ΔCt^) are shown relative to the median of values for normal breast epithelial tissues (N).

**Figure 2:**
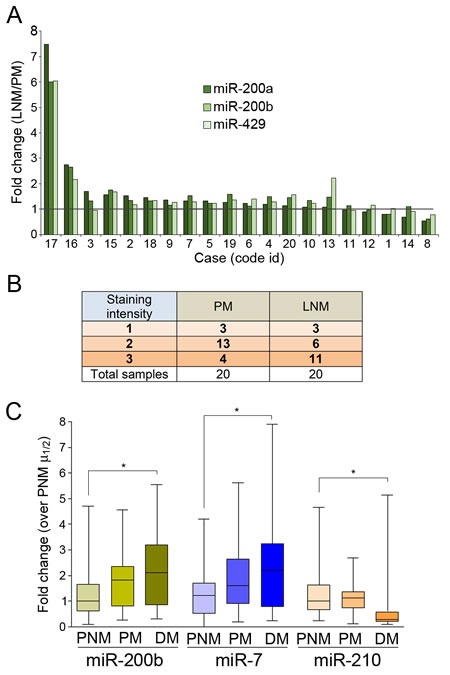
Upregulation of microRNA 200s in association with lymph node involvement in IDC **A.** miR-200s are frequently expressed at higher levels in lymph node metastases than matched primary tumors. Shown are ratios (fold-change) of qPCR values (n^- ΔCt^) for the indicated miRNAs in lymph node metastases (LNM) and their matched primary tumor (PM). **B.** Semiquantification of E-cadherin immunostaining of primary (PM) and matched metastatic (lymph node, LNM) ductal breast cancer samples. **C.** Blood levels of miR-200b and miR-7 tend to be higher, and levels of miR-210 lower, along metastatic progression in breast cancer patients. Reference probe-normalized values (n^- ΔCt^) are shown relative to the median of values for blood samples from patients with non-metastatic node-negative IDC (PNM μ_1/2_).

Focusing on lymph node spread as a readout for the metastatic potential of cancer cells, we sought to identify microRNAs (miRNAs) with dysregulated expression in the course of IDC metastatic progression. For this, a miRNA microarray expression screening was performed using tissue samples from 8 ductal carcinomas *in situ* (DCIS), 20 primary IDC without LN involvement (PNM), 20 primary IDC with regional LN involvement (PM), 20 LN metastases matched to the node-positive primary tumors (LNM) and 20 distant metastases (DM) (Supplementary Table 1) and a pool of 10 normal breast epithelial tissues (N). This analysis yielded miRNAs that were significantly upregulated in the transition from normal breast tissues to PM, including miR200a, miR-200b and miR-429 (Figure 1A). Another set of miRNAs, including miR-181a, miR-181b, miR- 210 or miR-7 was upregulated in DM relative to primary tumors (Figure 1A). qPCR confirmed that miR-200b, miR-7 and miR-210 are significantly upregulated from PNM to PM, while miR-7, miR-210, miR-181a and miR-181b were up-regulated in DM relative to node-negative tumors (Figure 1B). Furthermore, higher levels of miR-200a, miR-200b and miR-429 were expressed in a majority of LNM samples compared to their matched primary tumors (Figure 2A). Consistently, staining of these samples for E-cadherin, a hallmark of the epithelial reprogramming driven by miR-200s, revealed a generally stronger staining intensity in LNM cancer cells when compared to matched primary tumors (Figure 2B).

Although the primary mode of breast cancer dissemination is lymphogenous [5], if LN metastasis reflects a general propensity of breast cancer cells for metastatic spread, similar changes might be detected in the blood as circulating miRNAs. Because the tissue levels of miR-200b, miR-200c, miR-7, miR-10b, miR-148, miR-101, miR-30a*, miR-181a, miR-181b and miR-210 were significantly dysregulated during metastatic progression, we determined their levels in blood samples prospectively collected from 78 patients with a diagnosis of IDC (Supplementary Table 2). Patients with node-positive tumors at the time of diagnosis displayed higher miR-200b and miR-7 blood levels than patients with node-negative tumors (Figure 2C). Differences were also significant when comparing patients with distant metastases to those bearing only primary tumors (Figure 2C). The blood levels of miR-210 were significantly lower in patients with distant metastasis relative to those with node-negative primary tumors (Figure 2C). The remainder of the microRNAs analyzed did not show significant differences in their blood levels between any of the patient groups studied (not shown). The observed concordance between increased levels of miR-200b and miR-7 during metastatic progression in both tumor tissues and blood argue in favor of a carcinomatous origin of the circulating miRNAs.

### miR-200s promote luminal progenitor properties in tandem with tumorigenic and metastatic growth of MCF10CA1h cells

**Figure 3:**
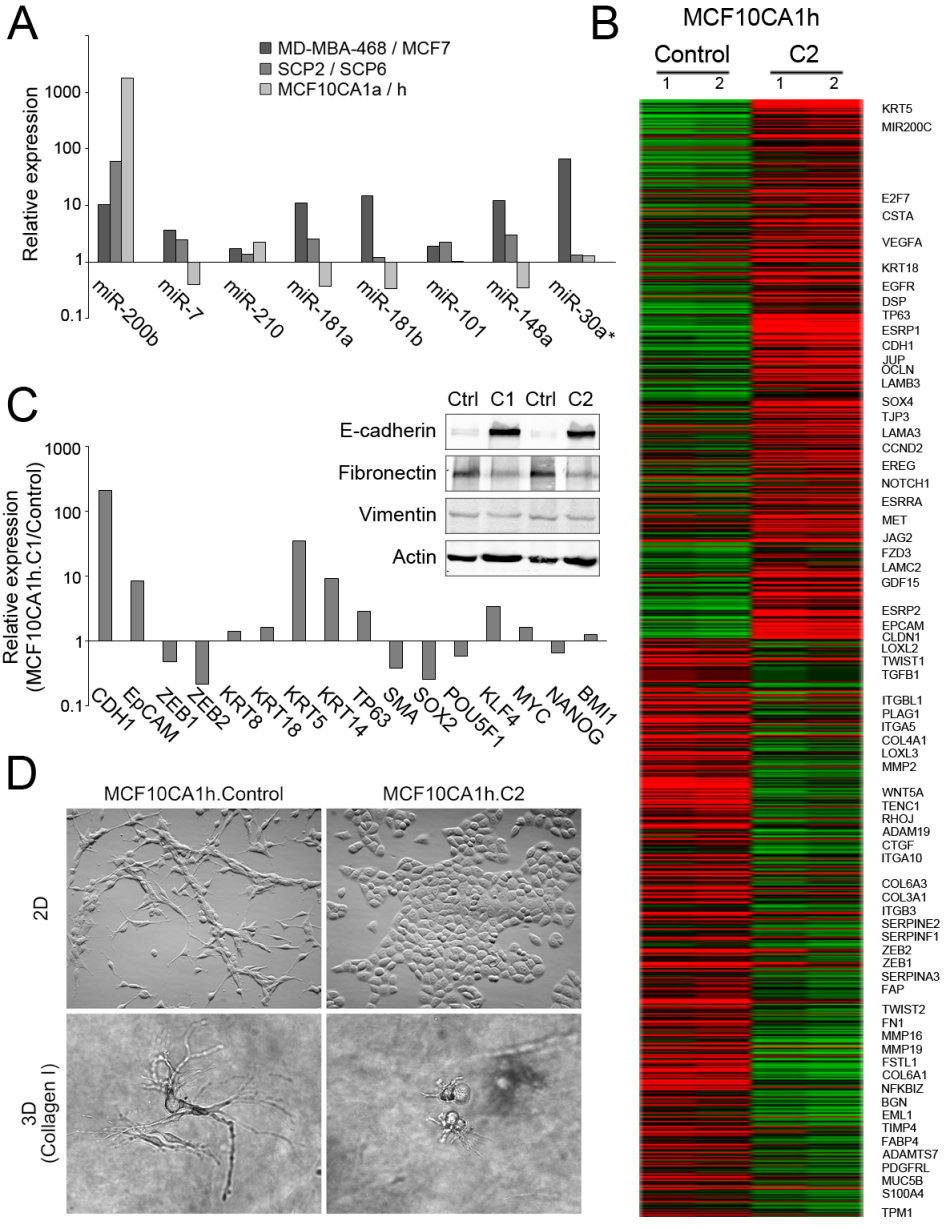
Phenotypic effects of miR-200s expression in MCF10CA1h cells **A.** Cell lines with stronger metastatic potential express higher levels of miR-200b. The MDA-MB-468 (highly metastatic) breast cancer cell line was compared to MCF7 cells (poorly metastatic), the MDA-MB-231-derived SCP2 clonal cell line (highly metastatic) to the SCP6 clone (significantly less metastatic) and the MCF10 breast epithelial-derived MCF10CA1a subpopulation (moderately metastatic) to the MCF10CA1h subpopulation (non-metastatic). Shown are ratios (fold-change) for the compared cell lines of reference miR-normalized qPCR values (n^- ΔCt^) for the indicated miRs. **B.** MiR-200s induce a mesenchymal-to-epithelial transition (MET) in MCF10CA1h cells, with upregulation of epithelial markers (CDH1, DSP and EPCAM), downregulation of mesenchymal markers (ZEB1, ZEB2, TWIST1, TWIST2, FN1) as well as upregulation of basal (KRT5, KRT14) and luminal (KRT8, KRT18) keratins. Heatmap of relative mRNA levels of epithelial & mesenchymal markers in MCF10CA1h.C2 *vs* MCF10CA1h.Control cells. Values for genes with significant differential expression between the two cell lines, determined by statistical analysis of microarrays (SAM) were submitted to hierarchical clustering and plotted in a heatmap. Shown on the right are the symbols of selected genes relevant in epithelial and mesenchymal gene programs. **C.** MET and basal marker induction in MCF10CA1h cells determined by qPCR (cluster 1 miR-200s) and Western blotting (cluster 1 - C1 - and cluster 2 - C2 - miR200s). MiR-200 induce a mesenchymal-to-epithelial transition (MET) in MCF10CA1h cells, with upregulation of CDH1 and EPCAM, downregulation of mesenchymal markers ZEB1, ZEB2, FN1 and ACTA2, as well as upregulation of basal (KRT5, KRT14) and luminal (KRT8, KRT18) keratins and downregulation of SOX2 (but not POU5F1, KLF4, MYC or BMI1). Shown are ratios (fold-change) for MCF10CA1h.C1 *vs*. MCF10CA1h. Control of RP18S-normalized qPCR values (n^- ΔCp^) for the indicated mRNAs **D.** Highly divergent morphologies of control and miR-200 (C2) - expressing MCF10CA1h cells grown as adherent cells on a plastic surface (top) or in 3-dimensional collagen I matrices (bottom).

The above results suggest that metastatic breast cancer cells express increased miR-200 levels as compared to primary tumor cells and thus are expected to display an enhanced epithelial phenotype. Although several studies have found an association between low miR200 levels with invasive cancers and poor prognosis, concluding that constitutive expression of miR-200s suppresses invasiveness and metastatic potential in xenotransplanation models [6, 7], more recent studies have reported that high-burden metastatic breast cancer cells express an epithelial gene program [8] and that miR-200s can play pro-metastatic roles [9–12]. To address this issue, we quantified the expression levels of miR200b in cancer cell models displaying differential metastatic potentials. Remarkably, two clonal lines derived from MD-MBA-231 basal-like cancer cells [13] presented a striking differential expression of miR-200b, with the highly metastatic SCP2 cells expressing 60-fold higher levels of miR-200b relative to poorly metastatic SCP6 cells (Figure 3A). Additionally, two subpopulations derived from the non-cancerous MCF10 cell line [14, 15], the moderately metastatic MCF10CA1a and the non-metastatic MCF10CA1h cells, also presented an unambiguous differential expression profile of miR200s, with MCF10CA1a cells expressing miR200b levels up to 3 orders of magnitude higher than those of MCF10CA1h cells (Figure 3A).

These MCF10 subpopulations are transformed variants of cells derived from non-tumoral mammary epithelial cells, with the MCF10CA1h subpopulation displaying a mesenchymal-like morphology and low anchorage-independent mammosphere growth potential relative to the more epithelial MCF10CA1a subpopulation [15]. We exploited these distinctive growth features to test the phenotypic consequences of the ectopic expression of miR-200s. Transduction of MCF10CA1h cells with miR-200 cluster 1 (C1; miR-200a, miR-200b, miR-429) or cluster 2 (C2; mir-200c, miR-141) induced an epithelial gene program with a strong upregulation of the epithelial genes, E-cadherin, EpCAM, and desmoplakin, together with a downregulation of the mesenchymal markers fibronectin, smooth muscle cell actin, TWIST1/2 or ZEB1/2 (Figure 3B-3C), accompanied with characteristic epithelial morphologies either grown on plastic or embedded in 3-D collagen I gels (Figure 3D). This gain in epithelial gene program and phenotype was accompanied with an inhibition of the capacity of miR-200-expressing MCF10CA1h cells to migrate in wound healing and invasiveness assays (Supplementary Figures 1 and 2).

**Figure 4:**
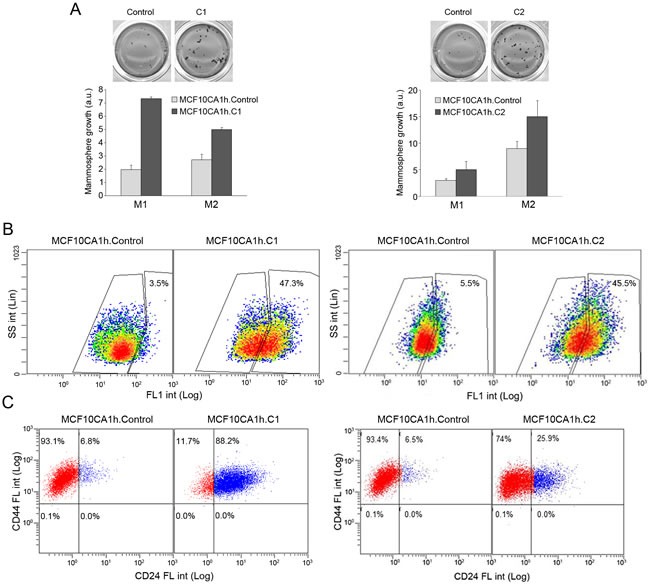
MiR-200s promote the mammosphere growth and ALDH activity of MCFCA1h cells **A.** miR-200-Cluster1 (C1; left) and miR-200-Cluster 2 (C2; right) microRNAs potentiate the mammosphere growth of MCF10CA1h cells. Cells (6 × 10^3^/well) were seeded in low-attachment plates and allowed to grow for 7 days (primary mammospheres, M1), disgregated and reseeded for secondary mammosphere formation (M2). Quantification was done after MTT staining and image analysis (*n* = 3). **B.** miR-200-C1 (left) and miR-200-C2 (right) strongly induce aldehyde dehydrogenase activity in MCF10CA1h cells. Cells were trypsinized and ALDH activity determined with the Aldefluor assay and flow cytometry. Windows for baseline fluorescence levels were established for cells incubated with the Aldefluor reagent and the ALDH inhibitor diethylaminobenzaldehyde (DEAB). **C.** miR-200-C1 (left) and miR-200-C2 (right) upregulate cell-surface CD24 in MCF10CA1h cells. Cells in suspension were incubated with Alexa Fluor 647-labeled anti-CD44 and Alexa Fluor-488-labeled anti-CD24 and analyzed by flow cytometry.

Importantly, transduction of miR-200s strongly enhanced the capacity of MCF10CA1h cells to form primary and secondary mammospheres (Figure 4A). This was accompanied with a striking 10-fold induction of aldehyde dehydrogenase (ALDH) activity (Figure 4B and Supplementary Figure 3). Further, miR-200s elicited a shift of MCF10CA1h cells from a CD44^high^CD24^low^ to a CD44^high^CD24^high^ cell surface haplotype (Figure 4C). While mammary epithelial stem cells are preferentially enriched in the CD44^high^CD24^low^ population [16], the CD44^high^CD24^high^ population encompasses more committed cell populations, including highly proliferating luminal progenitor cells and differentiated luminal cells [17], that likewise express high levels of ALDH activity [18, 19]. The upregulation of ALDH activity in miR-200-expressing MCF10CA1h cells was not accompanied with unamibugously enhanced resistance to drugs such as cis-Pt, etoposide, olaparib or docetaxel (Supplementary Figure 4). Taken together, these features are consistent with a miR200-mediated induction of a progenitor luminal state in mammary cells [17, 18, 20, 21].

**Figure 5:**
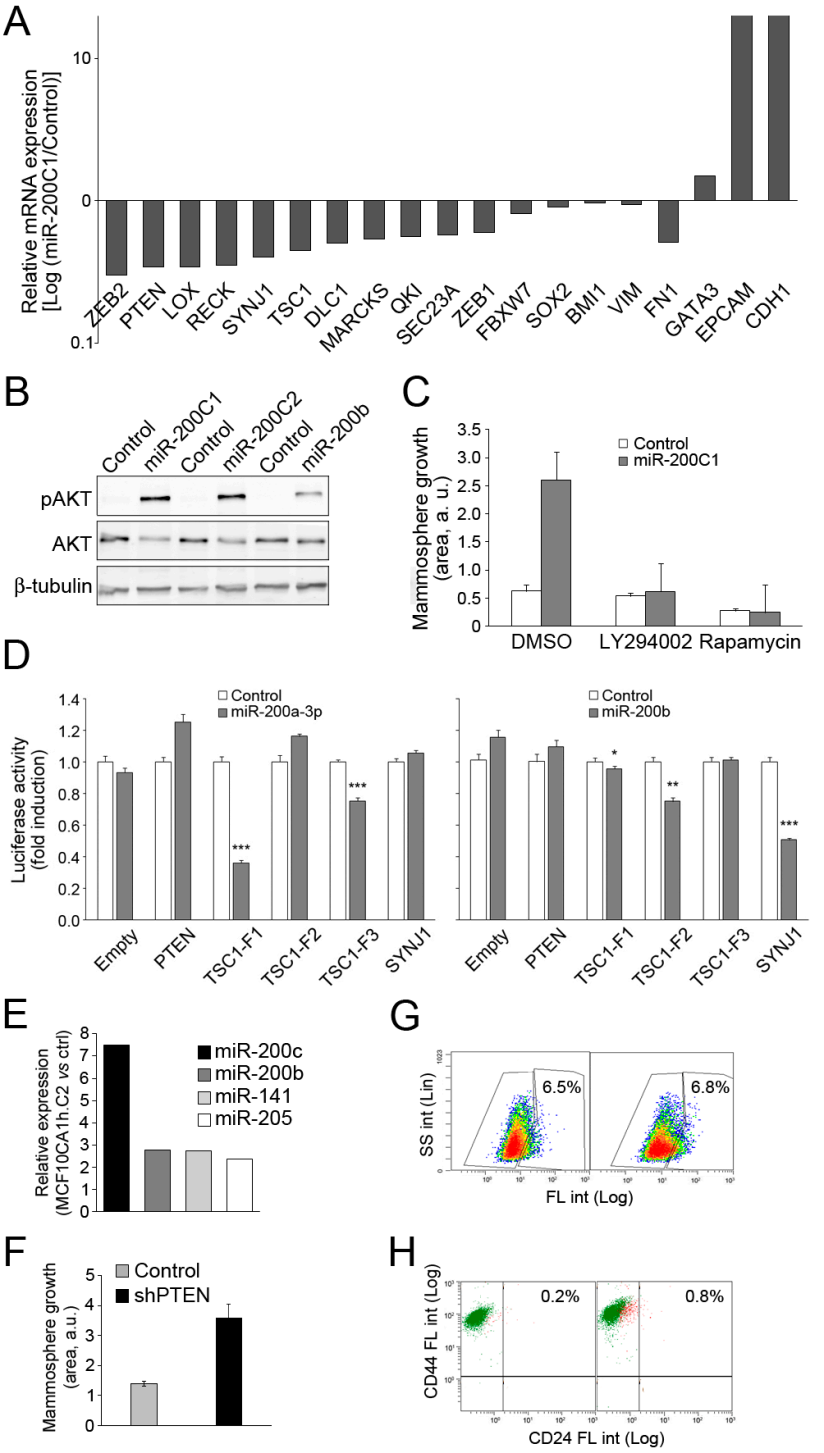
Activation of the PI3K-Akt signaling pathway by expression of miR-200 in MCF10CA1h cells **A.** Downregulation of known and predicted miR-200 target RNAs, determined by qPCR. As controls of miR-200 activity, the epithelial markers CDH1 and EPCAM are strongly upregulated, while the mesenchymal marker FN1 is downregulated. **B.** Induction by miR-200s of steady-state phosphorylation of Akt. Western blotting of total extracts of cells serum-starved for 24 h, probed with anti-pAkt (Ser473), total Akt or β-tubulin. **C.** Abrogation by LY294002 and rapamycin of miR-200(C1)-induced mammosphere growth of MCF10CA1h cells. **D.** The mTOR negative regulator TSC1 is a target of miR-200a and miR-200b, and the inositol-1,4,5-trisphosphate 5-phosphatase SYNJ1 is a target of miR-200b. Luciferase assays were run with constructs bearing the indicated 3’-UTR fragments. **E.** Lentivirally-mediated expression of miR-200-C2 in MCF10CA1h cells induces the expression of endogenous miR-205, as determined by microarray analysis. **F.** Knockdown of PTEN induces mammosphere growth in MCF10CA1h cells. **G.** Knockdown of PTEN fails to enhance ALDH activity in MCF10CA1h cells, as assessed by the Aldefluor assay. **H.** Knockdown of PTEN fails to induce cell surface expression of CD24, determined by flow cytometry.

**Figure 6:**
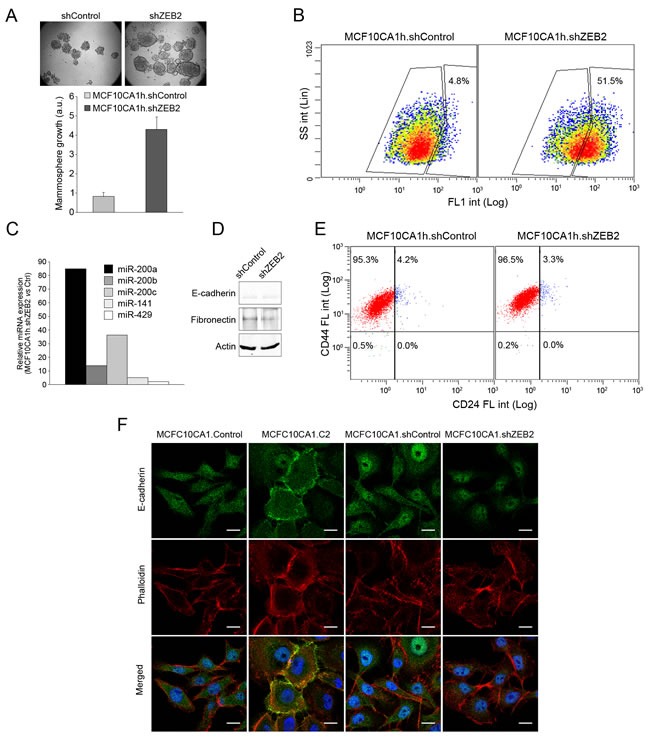
ZEB2 knockdown partially phenocopies miR-200 expression in MCF10CA1h cells **A.** Knockdown of ZEB2 potentiates the mammosphere growth of MCF10CA1h cells. Top, bright field images. Bottom, MTT quantifications (*n* = 3). **B.** Knockdown of ZEB2 induces ALDH activity in MCF10CA1h cells. **C.** Knockdown of ZEB2 induces the upregulation of endogenous miR-200 family microRNAs, as quantified by real-time PCR. **D.** Knockdown of ZEB2 fails to induce MET in MCF10CA1h cells, as determined by Western blotting for E-cadherin and fibronectin. **E.** Knockdown of ZEB2 fails to upregulate cell-surface CD24 in MCF10CA1h cells. **F.** Knockdown of ZEB2 fails to induce membrane-associated E-cadherin in MCF10CA1h cells, as assessed by immunofluorescence. Phalloidin-Alexa Fluor 568 was used to stain cortical cytoskeleton and Hoechst 33342 for DNA staining. Size bar, 10 μm.

To identify miR-200 targets that potentially control mammary cell fate determination, we quantified the expression levels in MCF10A1h cells a series of predicted miR-200 targets (http://www.microrna.org/microrna/home.do, http://www.targetscan.org/). Transcript levels for ZEB1, ZEB2, RECK, QKI, TSC1, SYNJ1 and PTEN were strongly downregulated upon expression of miR-200s (Figure 5A). Consistent with the observed downregulation of the PI3K-Akt negative regulator PTEN, an enhanced steady-state phoshporylation of Akt in MCF10CA1h.C1, MCF10CA1h.C2 or MCF10CA1h.200b cells relative to control cells was observed (Figure 5B). Furthermore, the gain in mammosphere formation conferred by miR-200s was completely abolished by the PI3K inhibitor, LY294002 or the mTOR inhibitor, rapamycin (Figure 5C), supporting a model wherein the miR-200-dependent activation of these signaling pathways promotes mammosphere formation. While novel miR-200a and miR-200b target status was demonstrated for the mTOR negative regulator, TSC1, PTEN was not directly targeted (Figure 5D), suggesting an indirect mechanism of downregulation by miR-200s [22]. In this regard, overexpression of miR-200 cluster 2 in MCF10CA1h cells caused an upregulation of miR-205 (Figure 5E), known to directly target PTEN, suggesting a mechanism whereby miR-200's induce the expression of miR-205, which in turn targets and downregulates PTEN. In support for a role of PTEN downregulation in the phenotype induced by mir-200, direct knockdown of PTEN in MCF10CA1h cells significantly enhanced mammosphere growth (Figure 5F). However, it failed to upregulate ALDH activity (Figure 5G) or the number of CD24^pos^ cells (Figure 5H). On the other hand, knockdown of ZEB2 in MCF10CA1h cells (Supplementary Figure 5) caused both a strong increase in mammosphere growth (Figure 6A) and ALDH activity (Figure 6B). Surprisingly, ZEB2 knockdown, despite prompting an upregulation of miR-200's (Figure 6C), did not completely phenocopy miR-200 expression, since it failed to engage a significant epithelial gene program (Figure 6D-6E) or upregulate CD24 in MCF10CA1h cells (Figure 6F).

These observations suggest that the acquisition of strong mammosphere growth induced by miR-200s, in part driven or reinforced by downregulation of ZEB2, is reinforced by the activation of the PI3K-Akt signaling pathway through indirect downregulation of PTEN. The latter represents a novel mechanism that may explain the significant growth and survival advantages conferred by the expression of miR-200 in MCF10CA1h and, possibly, breast cancer cells.

**Figure 7:**
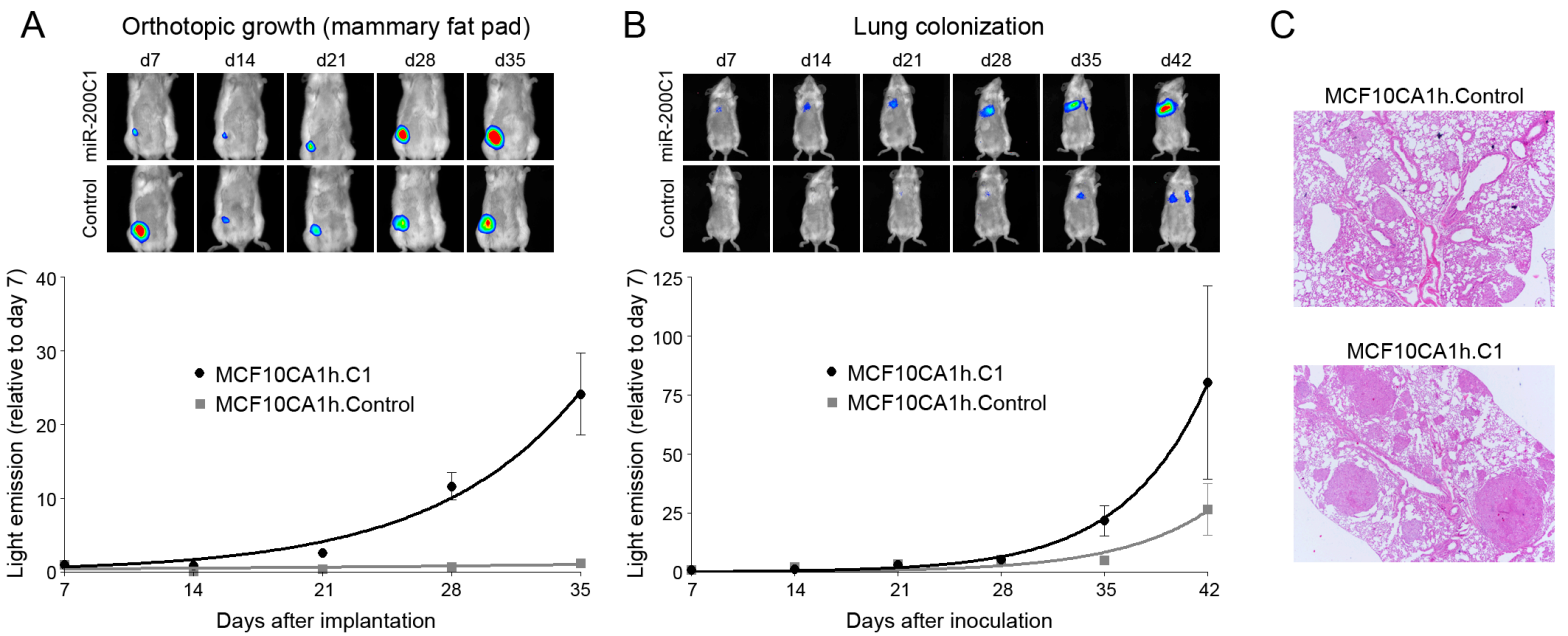
miR-200s potentiate the tumor growth and lung colonization of MCF10C1h cells in NOD-SCID mice **A.** Orthotopic implantation (cleared mammary fat pads) of luciferase-bearing MCF10CA1h.C1 cells, monitored by bioluminescence for 35 days (*n* = 8). **B.** Lung colonization assay after intravenous injection of luciferase-bearing MCF10CA1.C1 cells, monitored by bioluminescence for 42 days (*n* = 8). C. HE staining of representative tissue samples from lungs colonized by control and MCF10CA1h.C1 cells. Size bar, 100 μm.

After seven days of implantation in the cleared mammary fat pads of NOD-SCID mice, the growth of control cells stagnated while that of MCF10CA1h.C1 cells continued at an exponential rate (Figure 7A). Neither control nor miR-200-expressing MCF10CA1h cells produced detectable metastatic growth outside of their sites of implantation for the duration of local growth monitoring and up to two additional months of follow up after removal of the implanted tumors. However, intravenous inoculation of MCF10CA1h.C1 cells resulted in tumor colonization of the lungs at significantly higher rates than control cells with a significantly higher number of metastases than controls (mean: 7.6 *vs.* 2.9 per lung) (Figure 7B-7C), illustrating that enhanced metastatic colonization and growth can be uncoupled from local escape functions of tumor cells [23].

### miR-200s provide cues for the morphogenetic differentiation of MCF10CA1h cells

**Figure 8:**
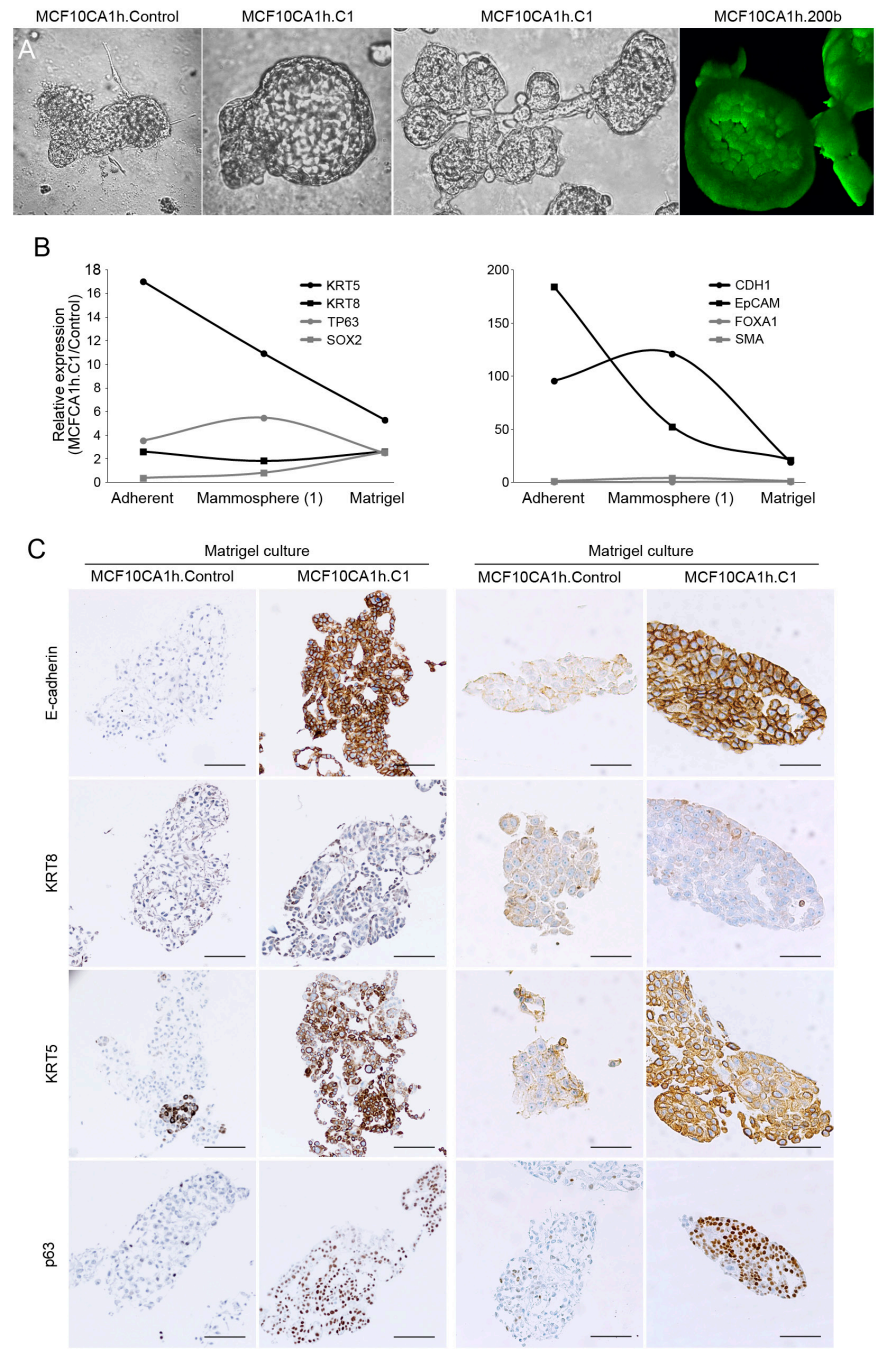
miR-200s promote the morphogenesis and differentiation of MCF10CA1h cells **A.** miR-200s promote the formation of pseudoalveolar (5-d culture) and complex tubuloalveolar (10-d culture) structures by MCF10CA1h cells grown in 5% Matrigel. The pseudoalveolar structures are hollow cavities formed by a single-cell layer as shown in 3-D reconstructions for GFP-expressing MCF10CA1.200b cells (right panel). Size bar, 100 μm. **B.** Modulation of miR-200 (Cluster1)-induced expression in MCF10CA1h cells of basal (KRT5, P63), luminal (KRT8, FOXA1), epithelial (CDH1, EPCAM) and self-renewal (SOX2) markers as a function of growth conditions (adherent, primary mammospheres, 5% Matrigel). mRNA levels were quantified by qPCR. Shown are ratios for values for MCFC10AC1.C1 *vs* control cells (fold-change). **C.** miR-200-Cluster 1 (left) or miR-200-Cluster 2 (right) induce an epithelial program (E-cadherin) and the concomitant upregulation of luminal (KRT8) and basal (KRT5) keratins *in vitro* (5% Matrigel 3D culture). Cells were formalin fixed and paraffin embedded and processed for immunostaining and diaminobenzidine-based detection with the indicated antibodies. Size bar, 100 μm.

The induction by miR-200s of breast luminal progenitor properties in MCF10CA1h cells prompted us to interrogate whether these cells could acquire further differentiated features under appropriate stimuli. When MCF10CA1h.C1 cells were grown in 5% Matrigel 3-D lattices, they formed highly organized branched tubular structures with multiple terminal hollow alveolar-like structures (Figure 8A), reminiscent of the complex structures formed *in vitro* from explanted normal mammary epithelial progenitor cells [24]. In contrast, control MCF10CA1h cells only formed amorphous or solid spherical structures (Figure 8A). Further, by co-expressing miR-200b and green fluorescent protein, we demonstrate that these cells contributed equally to both ductal- and alveolar-like structures (Figure 8A).

**Figure 9:**
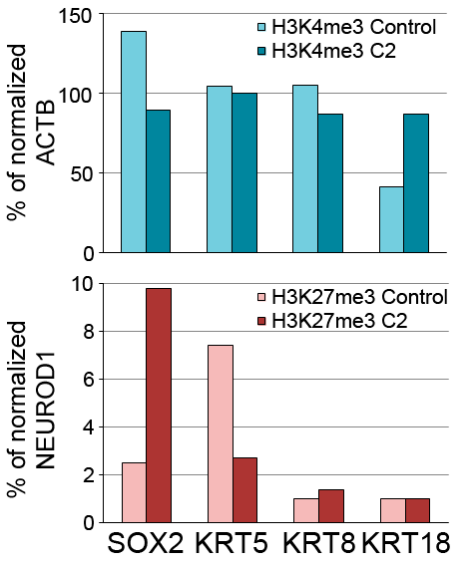
Chromatin immunoprecipitation to determine enrichment of histone markers H3K4me3 or H3K27me3 ChIP on promoter regions of SOX2, KRT5, KRT8 or KRT18 in control or miR-200 (Cluster 2)-expressing MCF10CA1h cells Shown are normalized input values for the test promoters relative to values for ACTB (H3K4me3 marks, associated with active transcription) or NEUROD1 (H3K27me3 marks, associated with repressed transcription).

In addition to the induction of an epithelial gene program, expression of miR-200s in MCF10CA1h cells elicited a marked upregulation of the basal keratin, KRT5 (Figure 8B-8C) and a more modest, but significant, induction of the luminal keratins, KRT8 and KRT18, as well as the basal marker, p63 (Figure 8B-8C). In concert with these changes, miR-200-expressing cells were enriched in the active transcription mark, histone H3K4me3, on the KRT18 promoter and depleted in the repressive mark, H3K27me3, on the KRT5 promoter (Figure 9), suggesting that the observed transcript modulation is at least partially attributable to a shift in transcriptional programs induced by miR-200s. miR200-expressing MCF10CA1h cells also showed a significantly higher proliferation index than control cells as determined by Ki67 staining (Supplementary Figure 6). These cells failed to express the luminal differentiation marker, estrogen receptor alpha, or the myoepithelial markers, myosin heavy chain. Remarkably, 3-D Matrigel cultures of MCF10CA1h.C2 cells, and more weakly MCF10CA1h.C1 cells, expressed the luminal differentiation master regulator, GATA3, which was not expressed in the absence of Matrigel (Supplementary Figure 6).

**Figure 10:**
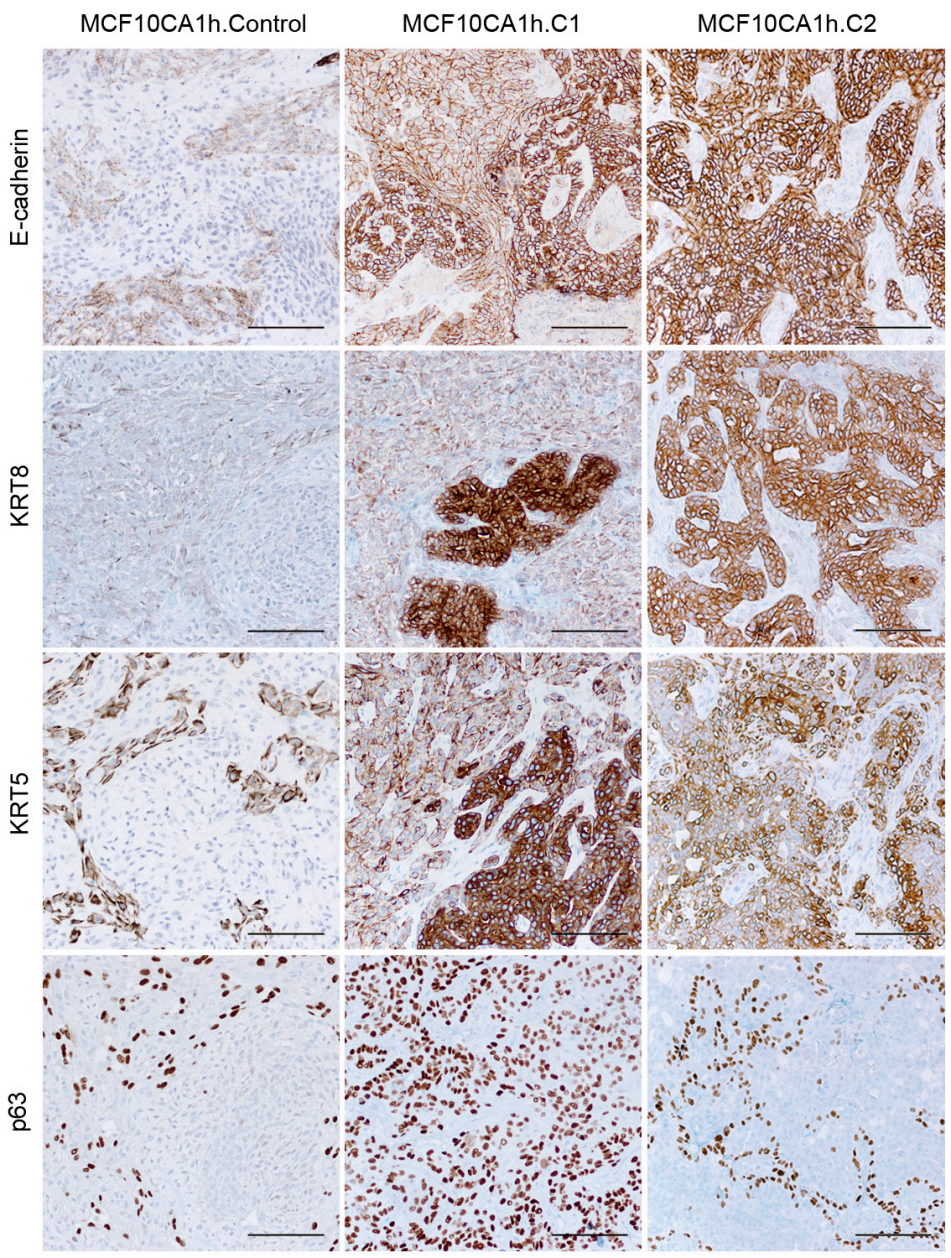
miR-200-Cluster 1 (left) or miR-200-Cluster 2 (right) induce an epithelial program (E-cadherin) and the concomitant upregulation of luminal (KRT8) and basal (KRT5) keratins *in vivo* Immunostaining with the indicated antibodies of paraffin embedded tissue samples from control and miR-200 (Cluster 1 or Cluster 2)-expressing MCF10CA1h cells grown as orthotopic implants in cleared mammary fat pads of NOD-SCID mice.

Tumors grown in mice upon orthotopic implantation of control MCF10CA1h cells displayed morphologically heterogeneous areas that included predominantly spindle-shaped and mesenchymal-like elements along with areas that included a more epithelioid appearance that failed to generate glandular structures (Figure 10). In contrast, tumors formed by MCF10CA1h.C1 and MCF10CA1h.C2 cells contained fields dominated by a more epithelioid appearance, including areas that had undergone morphological differentiation into gland-like structures (Figure 10). Similar to the phenotypes observed *in vitro*, the epitheloid components of these tumors showed intense staining for E-cadherin as well as luminal (KRT8) and basal (KRT5) cytokeratins (Figure 10). Further, whereas the mesenchymal marker, vimentin, was strongly and diffusely expressed in control tumors, it did so only in spindle-cell areas of MCF10CA1h-miR200 tumors (Supplementary Figure 7). Although GATA3 was focally expressed in epithelioid areas of MCF10CA1h.C2 tumors (Supplementary Figure 7), all tumors failed to express estrogen receptor-alpha or smooth muscle myosin heavy chain (not shown). Finally, p63 was diffusely expressed in spindle-cell areas of tumors from control cells and was highly expressed in periglandular cells in tumors from MCF10CA1h-miR200 cells (Figure 10).

To summarize, the upregulation of basal (KRT5, p63) and luminal (KRT8/18, GATA 3, CD24) markers by miR200 in these cells, together with the robust induction of ALDH activity and the epithelial markers, EpCAM and E-cadherin, as well as their capacity to undergo glandular differentiation upon orthotopic implantation in mice, reinforce the hypothesis that miR-200s drive the acquisition of luminal progenitor characteristics [17].

### The epithelial components of metaplastic breast cancer express high levels of miR-200 and luminal progenitor cell marker profiles

**Figure 11:**
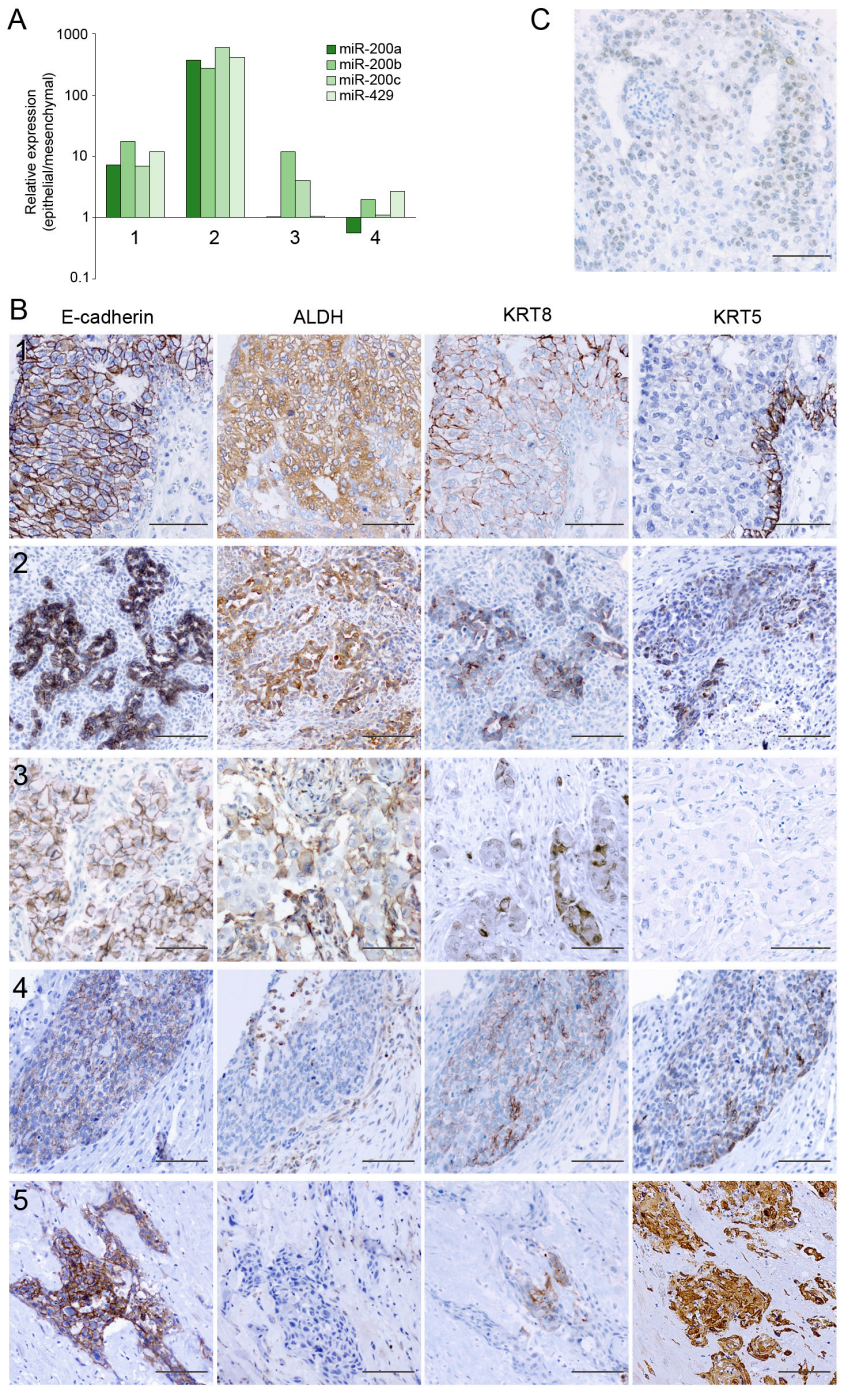
The epithelial components of metaplastic breast cancer can express high levels of miR-200s and markers of luminal progenitor cells **A.** Expression levels of miR-200a, miR-200b, miR-200c and miR-429 in enriched epithelial components relative to mesenchymal components of MBC samples, determined by qPCR. **B.** The epithelial components of MBC samples can co-express E-cadherin, KRT8, KRT5 and ALDH, determined by immunohistochemical analysis. Samples labeled 1-4 correspond to the samples with the same labels in (A). Size bar, 100 μm. **C.** Immunostaining of a metaplastic breast cancer case displaying weak but clear nuclear staining for GATA3. Size bar, 100 μm.

The morphological features of tumors formed in mice by MCF10CA1h-miR200 cells are reminiscent of metaplastic breast cancer (MBC) with spindle cell component [25]. As such, we hypothesized that the epithelial/glandular components of human MBC would express high levels of miR-200 and co-express luminal and basal markers. Given their histological and likely underlying genetic and epigenetic heterogeneity of these rare tumors [26], we restricted this analysis to five cases of the carcinosarcoma subtype, with well-delimited dual epithelial and mesenchymal components as determined by mutually exclusive expression of cytokeratins and vimentin. In 4 cases, we macrodissected the epithelial and mesenchymal components of the tumors, finding that the epithelial components in 3 out of the 4 tumors analyzed expressed significantly higher levels than the mesenchymal components of at least two of the five miR-200s quantified (Figure 11A).

Immunohistochemical analysis showed that KRT8 was expressed in the epithelial component of all five carcinosarcoma MBC cases (Supplementary Table 3). In all but one case, the same cellular areas co-expressed KRT5 (Figure 11B). In addition, ALDH was also strongly expressed in the epithelial components of 3 of the 5 cases studied, corresponding to those with a strong expression of KRT8 and weaker or focal expression of KRT5 (Figure 11B). As expected, E-cadherin was consistently and diffusely expressed in the epithelial components of all cases (Figure 11B) while estrogen receptor-alpha and HER2 were undetectable (not shown). Interestingly, one case with strong ALDH and KRT8 staining in the epithelial component also showed positive nuclear staining for GATA3 (Figure 11C). In sum, the epithelial components of MBC with double mesenchymal and epithelioid components (carcinosarcomas) express markers of breast luminal progenitor cells, including ALDH and double luminal-basal cytokeratins, coincident with high levels of miR-200s.

## DISCUSSION

In this study, we have identified sets of miRNAs that are dysregulated along the metastatic progression of IDC. Of these, only miR-200 and miR-7 showed the same trend in tissue samples and in blood, namely a statistically significant tendency for higher levels in distant metastatic than in node-positive primary cases and tumors (PM) and higher levels in PM than in node-negative primary cases and tumors. These concordances suggest that circulating miR-200 and miR-7 levels reflect the expression levels of these miRNAs in the tumors borne by the patients.

Previous reports have shown that miR-200s circulate at higher levels in association with metastasis in epithelial tumors [11, 27, 28] in consonance with reports demonstrating that circulating breast cancer cells with metastatic potential are predominantly epithelial [29] and that expression of miR-200s promotes the metastatic behavior of breast cancer cells [10–12]. However, a more broadly extended view is that miR-200s prevent metastasis through induction of an epithelial gene program in detriment of EMT and invasive properties of cells [6, 7]. Nevertheless, in addition to shifting the balance in favor of epithelial gene programs, miR-200s have been found to negatively regulate self-renewal gene networks, by targeting BMI1 or SOX2 and thereby limiting the growth of normal and tumor cells [30]. Indeed, we have observed a downregulation of SOX2 upon expression of miR-200s. However, this was accompanied with enhanced mammosphere growth, suggesting that expression of SOX2 does not play a significant role in the self-renewal properties of these cells. Likewise, our observations do not support a role for BMI1 in the maintenance of the growth characteristics of these cells, and thus alternative mechanisms must be invoked to explain the observed phenotypes. In this regard, the key roles found by others for miR-200s and an epithelial gene program in the generation of induced pluripotent stem cells with high self-renewal characteristics [31, 32] are in agreement with our observations that miR-200s promote, rather than counter, self-renewal (mammosphere growth) and metastatic properties of MCF10CA1h cells. Recent reports also support a metastasis-promoting function of miR-200 [10–12], in one case linked to its targeting of specific secretory pathways [10].

Our observations suggest a novel mechanism by which miR-200s promote self-renewal and the acquisition of progenitor cell characteristics, through activation of the PI3K-Akt-mTOR signaling pathway as a result of indirect downregulation of PTEN and direct targeting of TSC1. Although it has been reported by others that miR-200-family members bind to the 3’UTR of PTEN resulting in its downregulation [33], we have not found a direct interaction, which leads us to suggest an indirect mechanism. Relevantly, we have found that miR-205, a contrasted interactor and modulator of PTEN mRNA, is strongly upregulated in MCF10CA1h cells upon expression of miR-200’s. We thus propose that induction of miR-205 by miR-200's through mechanisms yet to be determined leads to a downregulation of PTEN and consequent constitutive activation of the PI3K-AKT-mTOR signaling axis. Downregulation of PTEN and activation of Akt and mTOR have been shown to be critical for proliferation and survival of CSCs and specifically, breast cancer stem cells [34], and an active PI3K/Akt/mTOR signalling axis is required for the maintenance of the undifferentiated properties of ESCs [35] and iPS cells [36]. Interestingly, the acquisition of a CSC-like phenotype by reprogramming differentiated luminal-like cells is associated with the activation of mTOR [37].

A further relevant and novel finding of our study is the induction by miR-200s of luminal progenitor-like features in MCF10CA1h cells, endowing them with a capacity for morphological differentiation, accompanied with the expression of markers associated with that stage in the differentiation of mammary epithelial cells. Thus, under the influence of miR-200’s, MCF10CA1h cells develop well-defined albeit imperfect (monolayered rather than bilayered) tubuloalveolar structures and engage limited proneness to display luminal differentiation, with weak expression of GATA3 when grown in Matrigel or *in vivo* but failure to express estrogen receptor. These observations suggest that complex breast morphogenetic events can take place in the absence of terminal differentiation of the two major breast epithelial lineages, that miR-200s promote this process and that environmental factors, yet to be identified, provide additional cues that drive the differentiation of miR-200-expressing breast epithelial cells along the luminal lineage.

The induction of high levels of ALDH activity, a CD44^high^CD24^high^ haplotype, enhanced mammosphere growth, alveolotubular morphogenesis *in vitro* and *in vivo*, and a proneness to luminal differentiation support our proposal that miR-200s induce a luminal progenitor phenotype in MCF10CA1h cells. In the context of our observations, prior evidences that constitutive expression of miR-200 in breast stem cells causes attrition of luminal progenitor cells thus preventing luminal differentiation and correct breast morphogenesis [30] may indicate that the promotion by miR-200s of luminal progenitor phenotypes occur within defined developmental windows and that prolonged expression cripples normal progenitor cells in physiological contexts. Our observation of high levels of expression of miR-200s in breast cancer cells with aggressive phenotypes or in MCF10CA1a cells indicate that the compromise in viability imposed by constitutive expression of miR-200s in normal breast stem cells can be overcome by factors that drive spontaneous (neoplastic) or experimentally induced immortalization. The precise correspondence of the MCF10CA1h cell line to a physiological stage along the mammary epithelial lineage has not been established, and therefore it is difficult to propose whether miR-200s drive these cells from a less differentiated to a more differentiated stage or in the converse direction. The failure of these cells to undergo full terminal differentiation after miR-200s expression is more likely related to specific constraints pertaining to MCF10 cells, either inherent in the original cells or acquired through the generation of different immortalized variants and subpopulations [14, 15] than to a differentiation block imposed by miR-200s [30].

Our analysis of metaplastic breast cancer samples indicates that tumor cells expressing epithelial programs and miR-200s can co-express luminal and basal keratins and ALDH. Co-expression of luminal and basal keratins in MBC has been reported previously and interpreted as a reflection of the cells of origin of these tumor cells being bipotent stem cells or myoepithelial cells [38–40]. Although such interpretations may apply to some subtypes of MBC, they may not suit the carcinosarcoma-type cases, since they fail to consider that normal breast epithelial stem cells express low levels of ALDH [18] and that myoepithelial cells do not express luminal keratins [17]. In light of the evidences presented herein for the MCF10CA1h cell model, our findings of co-expression of luminal and basal keratins and ALDH suggests that the epithelial neoplastic components of a subset of MBC may have originated in cells expressing an early progenitor luminal phenotype but retaining bipotent features. Evidences suggest that most other types of breast cancer originate from luminal progenitor cells [17, 41], with a minority of tumor types, including claudin-low and some metaplastic tumors [26], possibly originating from less differentiated cells that might correspond to mammary stem cells [17]. The epithelial components of MBC may harbor sufficient phenotypic plasticity to give rise to mesenchymal (and other metaplastic) neoplastic components through the engagement of EMT in response to epigenetic cues or genetic mutations [42, 43]. In addition, based on our observations with MCF10CA1h cells, we propose that the more intrinsically aggressive components of MBC are the epithelial components, rather than the mesenchymal components. Indeed, metastatic samples from MBC tend to display hallmarks of epithelial differentiation [44] and subtypes with a higher representation of epithelial components have been reported as associated with higher rates of distant metastasis and worse outcomes than those with a greater representation of mesenchymal components [45].

In conclusion, we propose that microRNAs of the miR-200 family promote traits of highly proliferative breast luminal progenitor cells and may contribute to exacerbate the growth and metastatic properties of transformed breast epithelial cells, including those present in histological variants such as metaplastic carcinomas.

Supporting Information is available online, containing Supplementary Tables and Figures

## MATERIALS AND METHODS

### Sample procurement and processing

All patient samples were procured through the Hospital Clínic-IDIBAPS Biobank. Formalin-fixed and paraffin-embedded (FFPE) samples from invasive ductal carcinomas were selected on the basis of estrogen receptor (ER), progesterone receptor (PR) and HER2 expression. Also, normal breast epithelial tissues from patients undergoing reductive mammoplasty were collected (Supplementary Table 1). Samples with > 70% tumor epithelial enrichment were macrodissected to minimize stromal and lymphocytic components. Blood samples were collected from untreated breast cancer patients at diagnosis and 6 healthy women with negative mammographies (Supplementary Table 2).

### Immunohistochemical analyses

Tissue microarrays (TMA) were built from paraffin-embedded samples bearing 3-μm thick, 1-mm diameter triplicate cores. Samples included those used in the initial microRNA screening and subsequent analyses, and additional infiltrating carcinomas and lymph node metastases as well as 5 metaplastic breast carcinomas of the carcinosarcoma type. Samples were deparaffinized and rehydrated (PTLink module, Dako Omnis) prior to antigen retrieval (97 °C for 20 min with the EnVision™ FLEX low and high pH Target Retrieval Solutions, Dako Omnis, or 95° for 30 min with the Cell Conditioning Solution, Ventana), followed by incubation with primary antibodies (Supplementary Table 4), incubation with polymer-peroxidase-conjugated secondary antibody and developed with diaminobenzidine. Slides were counterstained with haematoxylin, dehydrated and coverslipped. The reaction specificity was ascertained by the absence of staining when using a non-specific isotype-matched primary antibody. Nuclear immunostaining for ERα, PR and GATA3 was scored according to intensity (I; 0, 1, 2 and 3) and percentages of positive cells. Hscores ≥ 2 were considered as positive staining. Membrane staining for HER2 was assessed as 0, 1, 2 and 3, following recommendations of the American College of Pathologists [46]. Only cases with HER2 score equal to 3 were considered HER2(+). Primary tumors were classified according to the expression of hormone receptors and HER2. Cases were considered as “luminal” with positive ER and/or PR (≥ 10% positive cells) and negative HER2 (0, 1). Membrane E-cadherin expression was scored according to the product between the intensity coefficient (0, negative; 1, low; 2, moderate; 3, strong) and the frequency of positivity coefficient (0, no stained cells; 1, 1-9%; 2, 10-49%; 3, 50-79%; 4, 80-100%) and classified as follows: 0, negative score; 1+, score 1-4; 2+, score 5-8; 3+, score 9-12 [47].

### RNA isolation, reverse transcription and real-time RT-PCR of cell culture samples

For RNA extraction of adherent cells, cells were grown to 70-80% confluence and lysed directly on the plate with Qyazol lysis reagent. Mammospheres were collected by gentle centrifugation and resuspended with Qyazol. 3D structures were recovered from Matrigel using the non-enzymatic solution Matrisperse (Cultek), following manufacturer's instructions, and resuspended in Qyazol after gentle centrifugation. RNA was isolated with the miRNeasy Mini kit (Qiagen). cDNA was synthesized with the HighCapacity cDNA Reverse Transcription Kit (Applied Biosystems). Real-time quantitative PCR assays were performed on a LightCycler 480 instrument (Roche) and analyzed with the LightCycler 480 Software release 1.5.0. The Universal Probe Library system (UPL) (Roche) was used to quantify transcripts. Probes and sequences are shown in Supplementary Table S5. RN18S5 amplification levels were used as an internal reference, and relative transcript quantification determined by the ∆∆Cp method.

### MicroRNA microarray analysis

Total RNA was extracted from FFPE tissues with the miRNeasy FFPE kit (Qiagen), labeled using the Agilent miRNA Labeling and Hybridization Kit (Agilent Technologies Incorporated, Santa Clara CA, USA), hybridized to Agilent Human miRNA microarrays (V12.0, Agilent), scanned on an Agilent scanner and feature extracted using Agilent Feature Extraction Software (version 10.5.1.1). Quantile normalization was performed using the “normalize.quantiles” function from the “affy” R package in Bioconductor (http://www.bioconductor.org) and invariant and highly expressed microRNAs selected [48]. Raw data for this analysis are accessible at Gene Expression Omnibus, http://www.ncbi.nlm.nih.gov/geo/ (GSE86995).

### MicroRNA quantitative PCR

Total RNA was retrotranscribed with the Universal cDNA Synthesis kit (Exiqon). Mature microRNAs were detected using the ExiLENT SYBR Green Master Mix (Exiqon) and specific LNA^™^ primers. miR-16 and let7a were used as reference microRNAs. Real-time quantitative PCR assays were performed on a StepOnePlus instrument (Life Technologies). Relative quantifications were assessed by the ∆∆Cp method. Normalized values were used in comparative analysis between categories of samples using either parametric (*t*-test) or non-parametric tests (Mann-Whitney). For quantification of miR in blood, total RNA was isolated from 2.5 mL of blood collected in PAXgene Blood RNA tubes (Qiagen), retrotranscribed and microRNA levels determined by real-time PCR as above, using miR-16 and miR-103a-3p as references.

### mRNA microarray analysis

Total mRNA was isolated and processed for hybridization on Human Gene ST 2.1 strips (Affymetrix). Signals were fitted with a probe-level model and expression values were calculated and log_2_ transformed using a robust multi-array average (RMA) [49]. Probes with ≥ 2-fold change in intergroup comparisons were selected for hierarchical clustering analysis and heatmap plotting [50].

### Cell culture

MCF10CA1a and medium MCF10CA1h cells were maintained in DMEM/F12 supplemented with 5% horse serum, 10 μg/mL insulin (Sigma-Aldrich), 20 ng/mL epidermal growth factor (Life Technologies), 0.5 μg/mL hydrocortisone (Sigma-Aldrich), and 50 ng/mL cholera toxin (Sigma-Aldrich). The human breast carcinoma cell lines MDA-MB-468 and MCF7, the MDA-MB-231 cell derivatives SCP2 and SCP6 and HEK293T cells were cultured in DMEM culture medium (PAA) containing 10% fetal bovine serum and penicillin/streptomycin. Cells were grown at 37 °C in a 5% CO2 atmosphere.

### Collagen I 3-D culture

Type I collagen was isolated from mouse tail tendons as described previously [51] and dissolved in 0.2% acetic acid at a final concentration of 2.7 mg/mL Before gelation, the collagen solution was mixed with 10× minimum essential media (MEM) and 0.34 N NaOH at a ratio of 8:1:1 at 4 °C, with MCF10CA1h control and MCF10CA1h. C2 cells (1-5 × 10^6^) suspended in 1 mL of this mixture. The carcinoma cell-collagen mixtures were incubated for 1 h at 37 °C to allow for gelation and culture media (MEM supplemented with 10% FCS) added atop the gel.

### Wound-healing assay

Cells were seeded in 24-well plates (Corning) at 2 × 10^4^/well in DMEM:HAM medium supplemented with 5% horse serum (HS). After reaching 80% confluence, the medium was replaced with fresh medium devoid of serum containing 0.5% (w/v) mitomycin C (Sigma). After 1 h of treatment, medium was replaced with fresh medium supplemented with 0.5% HS and approximately 1-mm wide wounds produced in the confluent monolayer. Wounds were imaged at 0 h, 12 h, 24 h and 48 h and analyzed with the aid of Image J. At least three wounds per condition were scored.

### *In vitro* invasiveness assay

Transwell chambers (Costar) with 8-μm diameter pore membranes were coated with growth factor-reduced Matrigel (BD Biosciences) at 410 μg/mL. Cells were serum-deprived for 24 h, detached, resuspended in medium supplemented with 1% BSA/0.5% FBS and then seeded (1.5 × 10^5^/well in 24-well plates) onto the pre-coated Transwell inserts, with the lower chamber containing medium supplemented with 0.5% FBS. After 24 hours, cells migrating to the lower chamber were collected by detachment with trypsin-EDTA, washed with PBS, and fluorescent cells scored in a Coulter Epics XL instrument (Coulter Electronics, Luton, UK). Each condition was done in quadruplicate.

### Cytotoxicity assay

Cells were seeded in 96-well plates (Corning) at 1.5 × 10^3^ cells/well, allowed to attach overnight and exposed to varying concentrations of drugs for 96 h. After treatment, 10 μL of MTT ((3-(4,5-dimethylthiazol-2-yl)-2,5-diphenyltetrazolium bromide) solution (Sigma) were added and incubated at 37 °C for 3 h. Crystals were solubilized with 100 μL of 0.08 M HCl/isopropanol by shaking for 30 min in the dark, and absorbance recorded at 570 nm. Each condition was done in quintuplicate.

### Mammosphere growth

MCF10 variant cell lines were obtained from the Karmanos Cancer Institute (MTA with Y. Kang). Cells were grown in DMEM:F12 medium supplemented with B27, 20 ng/mL EGF, 20 ng/mL bFGF (Life Technologies), 4 μg/mL heparin, 5 μg/mL insulin and 0.5 mg/mL hydrocortisone (Sigma-Aldrich). Cells (10^4^/mL) were plated per well 24-well in ultralow attachment plates (Corning). For serial passages, mammospheres were centrifuged at 800 rpm, dissociated with 0.05% trypsin, 0.53 mM EDTA, sieved through a 70-μm sieve, analyzed microscopically for single-cellularity and replated. Colonies were stained with MTT, images acquired and areas quantified with Image J.

### Differentiation culture conditions

3D cultures were performed as previously described [52]. Primary mammospheres were dissociated and grown in DMEM:Ham's F-12 medium with 5% Matrigel (Corning), 5% 24-well, 10 μg/mL insulin, 0.5 μg/mL hydrocortisone, 10 μg/mL cholera toxin, 10 ng/mL EGF, and 1× Pen/Strep. Single cell suspensions were plated on Matrigel-precoated plates at a density of 40,000 cells/mL and allowed to form three-dimensional structures for 10-14 days.

### Lentiviral production and transduction

For the generation of stable miR-200 overexpressing cell lines, pMSCV-puro with our without the genomic fragment harboring miR-200 cluster 1 (miR-200a/200b/429), pMSCV-hygro with our without the genomic fragment harboring cluster 2 (miR-200c/141), were co-transfected with pVSVG into the retrovirus packaging cell line PG13 (Clontech, Mountain View, CA, USA) using X-tremeGENE9 (Roche). Supernatants were collected for the following 48 h and filtered through 0.45 μm methylcellulose filters (Millipore). Virus-containing supernatants were used to infect target cells in the presence of polybrene (8 μg/mL; Sigma-Aldrich), and selected with 1 μg/mL puromycin (Sigma-Aldrich) or 50 μg/mL hygromicin B for 7 days. To overexpress hsa-miR-200b, the pCDH-CMV-MI0000342-EF1-copGFP lentiviral vector and its control were purchased from System Biosceinces (Paolo Alto, CA). Each plasmid was co-transfected in HEK293T cells with pVSVG and pCMVΔR8.91 (Clontech, Mountain View, CA, USA) using X-tremeGENE9 (Roche). Supernatants were collected for the following 48 h, filtered through 0.45 μm methylcellulose filters (Millipore) and were used to infectthe target cells in the presence of polybrene (8 μg/mL; Sigma-Aldrich). At 72 hours after infection, successful gene transfer was confirmed by visualization of GFP by fluorescence microscopy and positive cells were enriched by fluorescence-activated cell sorting (FACS) (MoFlo, Beckman Coulter).

For the generation of stable ZEB2 and PTEN knockdown cell lines, after screening for the most effective specific knock down shRNAs (5 distinct target sequences per mRNA), pLKO.1-Puro plasmids for control (shC002), ZEB2- or PTEN-targeting shRNAs (TRCN0000013528 and TRCN0000002745, respectively) (Sigma-Aldrich) were co-transfected in HEK293T cells with pVSVG and pCMVΔR8.91 (Clontech, Mountain View, CA, USA) using X-tremeGENE9 (Roche). Supernatants were collected for the following 48 h and filtered through 0.45 μm methylcellulose filters (Millipore. Target cells were transduced with the supernatans in the presence of polybrene (8 μg/mL; Sigma-Aldrich), and selected with 1 μg/mL puromycin (Sigma-Aldrich) for 5 days.

### Flow cytometry

Aldehyde dehydrogenase activity was detected with the Aldefluor kit (Stem Cell Technologies) used according to manufacturer's protocol and analyzed by flow cytometry on a Gallios Flow Cytometer instrument (Beckman Coulter). For cell surface immunophenotyping, cells were detached with 0.25% trypsin/ 0.1% EDTA, washed and incubated with primary antibodies CD44 (Alexa Fluor 647, anti-mouse/human, 1:4000 dilution, BioLegend) and CD24 (Alexa Fluor 488, anti-human, 1:20 dilution, BioLegend) in PBS/3% normal goat serum for 30 min in a shaker at 4 °C, washed and analyzed by flow cytometry.

### Western blotting

Samples were resuspended in Laemmli buffer (100 mM dithiothreitol, 50 mM TrisCl pH 6.8, 2% SDS, 0.1% bromophenol blue, 10% glycerol) and boiled for 5 min. Equal amounts of protein were resolved by SDS-PAGE. After electrophoresis, proteins were transferred onto fluorescent-PVDF membranes (Immobilon-FL, Millipore) for 2-4 h. Blots were washed, blocked with blocking buffer (Odyssey, LI-COR Biosciences, Lincoln, NE, USA), incubated overnight with primary antibody diluted in blocking buffer-PBS (or TBS)-Tween (0.1%) (1:1), washed in PBS-T (or TBS-T) and incubated for 1 h with IRDye fluorescent secondary antibody (LI-COR). After final washes in PBS-T (or TBS-T), the membranes were scanned using the Odyssey Infrared Imaging System (LI-COR). Antibodies to the following antigens were used: E-cadherin (1:8000) (BD Bioscience), FN1 (1:2000) (Sigma), N-cadherin (1:1000) (BD Bioscience), Vimentin (clone V9, 1:2000) (Labvision) and β-tubulin (1:2000) (Sigma).

### 3’UTR luciferase reporter assays

psiCHECK2-PTEN 3’UTR construct was obtained from Addgene (Plasmid 50936). A plasmid containing the 3’UTR of SYNJ1 was generated by cloning a 3’UTR fragment of 628 bp using the Zero Blunt PCR cloning kit (Invitrogen). Due to size constraints, TSC1 3’UTR was cloned as three different fragments (Fragment #1:483bp; fragment #2: 941bp; fragment #3: 368bp) into the pCR Blunt vector. SYNJ1 and TSC1 3’UTR fragments were subcloned into psiCHECK-2 (Promega) using XhoI and NotI restriction sites. The primers used are listed in Supplementary Table S6. Reporter assays were performed as follows: HEK293T were transduced with lentiviral particles carrying either pmiR-empty or pmiR-200b. Seventy-two hours post-infection, cells were seeded into 96-well plates and transfected with 50 ng of the indicated 3’UTR reporter vectors for an additional 24 h (*n* = 4 per condition). Luciferase activity was measured using the Dual-Glo Luciferase AssaySystem (Promega). Renilla luciferase activity was normalized to corresponding firefly luciferase activity and plotted as a percentage of the control.

### Chromatin immunoprecipitation

Adherent cells were fixed in 1% formaldehyde for 10 min, quenched with 0.125 M fresh glycine for 5 min, washed twice with PBS, lysed (1% SDS; 10 mM EDTA pH8.0; 50 mM Tris-HCl pH 8.1, with protease inhibitors), and samples were kept on ice for 20 min. Cell lysates were sonicated in a Branson 450 sonicator (5 cycles of 20 seconds at 30% amplitude) to yield 200-500 bps chromatin fragments. Chromatin was purified by centrifugation at 13,200 rpm at 4 °C for 30 min, precleared with protein A agarose during 30 min and 25 μg of chromatin were immunoprecipitated with 5 μg of one the following antibodies: H3K27me3 (Millipore 07-449), H3K4me3 (Abcam ab8580) or nonspecific rabbit IgGs (Diagenode C15410206). Antibody-chromatin complexes were recovered with magnetic beads (Magna ChIP™ Protein A Magnetic Beads (Millipore 16-661) and immunocomplexes were washed once with TSE I (0.1% SDS; 1% Triton-X100; 2 mM EDTA pH 8.0; 20 mM Tris-HCl pH 8.1; 150 mM NaCl), TSE II (0.1% SDS; 1% Triton-X100; 2 mM EDTA pH 8.0; 20 mM Tris-HCl pH 8.1; 500 mM NaCl), TSE III (0.25 M LiCl; 1% NP-40; 1% Sodium Deoxicholate; 1 mM EDTA pH8.0; 10 mM Tris-HCl pH 8.1) and twice with TE (10 mM Tris-HCl, 1 mM EDTA). Crosslinking was reversed by overnight incubation at 65 °C in elution buffer (1% SDS, 0.1 M NaHCO_3_). DNA was purified by phenol-chloroform extraction followed by ethanol precipitation. Enrichment of target regions was determined by qPCR in a Lightcycler 480 instrument (Roche) using the primers listed in Supplementary Table S7. A fraction of input was used for the quantification of the immunoprecipitated material with respect to the total starting chromatin and the latter values for nonspecific IgG subtracted from values for specific antibodies. A region in the ACTB promoter was used as a control for the enrichment of H3K4me3 marks, and a region in the NEUROD1 promoter as a control for the enrichment of H3K27me3 marks. Percent input values for test promoter regions were normalized against these two controls to calculate relative enrichments in these two histone marks.

### *In vivo* tumorigenic and lung colonization assays

Cells were transduced with pCMV-GFP/luc for the constitutive co-expression of the firefly luciferase gene and GFP and selected by FACS. Female SCID-NOD mice (8-10 weeks) were injected with 50 μL of 2 × 10^6^ cells suspended in 50% Matrigel/PBS into cleared abdominal mammary fat pads. Tumor growth was monitored after intraperitoneal injection of 150 mg/kg D-luciferin (Caliper Life Science) and imaging in an ORCA-2BT instrument (Hamamatsu Photonics). To assess lung colonization, 5 × 10^5^ cells in 150 μL were injected in the tail vein of mice.

### Study approval

All protocols involving patient selection and sample procurement complied with Spanish laws regarding data protection and written informed consent and were approved by the Hospital Clinic - IDIBAPS Ethics Committee and Review Board. All animal procedures were reviewed and approved by the Institutional Animal Experimentation Ethics Committee (CSIC).

## SUPPLEMENTARY MATERIALS FIGURES AND TABLES







## Abbreviations

ALDH: aldehyde dehydrogenase
CSC: cancer stem-cell
DCIS: ductal carcinoma *in situ*
DM: distant metastases
ESC: embryonic stem cells
IDC: invasive ductal carcinoma
iPSC: induced pluripotent stem cells
LN: lymph node
LNM: lypmh node metastases
MBC: metaplastic breast cancer
miRNAs: microRNAs
N: normal breast tissue
NOD-SCID: non-obsese diabetic severe combined immunodeficient
PM: primary metastatic tumor
PNM: primary non-metastatic tumor
