# Supplementary material for: MicroRNA-200, associated with metastatic breast cancer, promotes traits of mammary luminal progenitor cells
Source: Oncotarget. 2017 Sep 7;8(48):83384–406. doi: 10.18632/oncotarget.20698 (PMC5663523; doi:10.18632/oncotarget.20698)
Supplement: Supplementary file 1 [file oncotarget-08-83384-s001.pdf]

MicroRNA-200, associated with metastatic breast cancer, promotes traits of mammary luminal progenitor cells

SUPPLEMENTARY MATERIALS

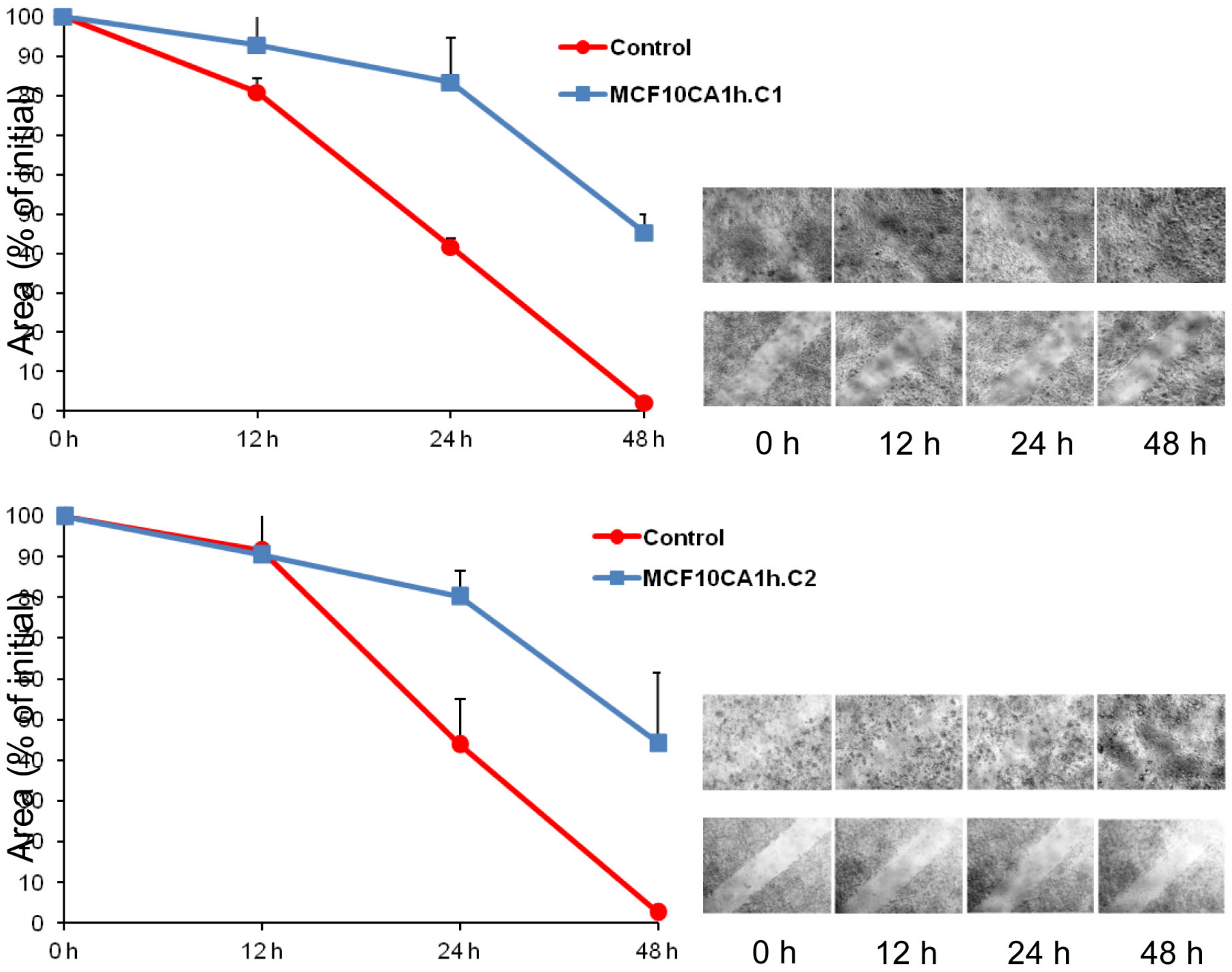

Supplementary Figure 1: miR-200 expression inhibits the migration of MCF10CA1h cells. Cells were plated to reach 100% confluence and scratches were made across the resulting monolayers. Images were captured at the indicated time points and the surface area between the advancing cell fronts quantified with the aid of Image J.

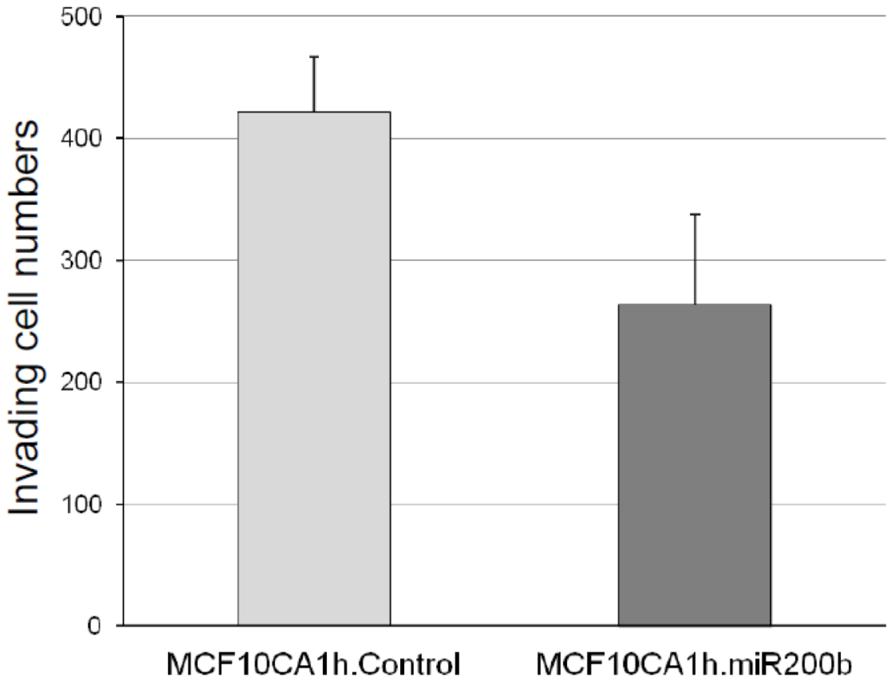

Supplementary Figure 2: Mir-200b expression inhibits the invasion of MCF10CA1h through Matrigel. Cells were placed in the upper chamber of Boyden chambers (Transwell, Corning) pre-coated with Matrigel. Both control and miR-200b-expressing cells express EGFP, and their migration through Matrigel into the lower chamger was quantified by flow cytometry.

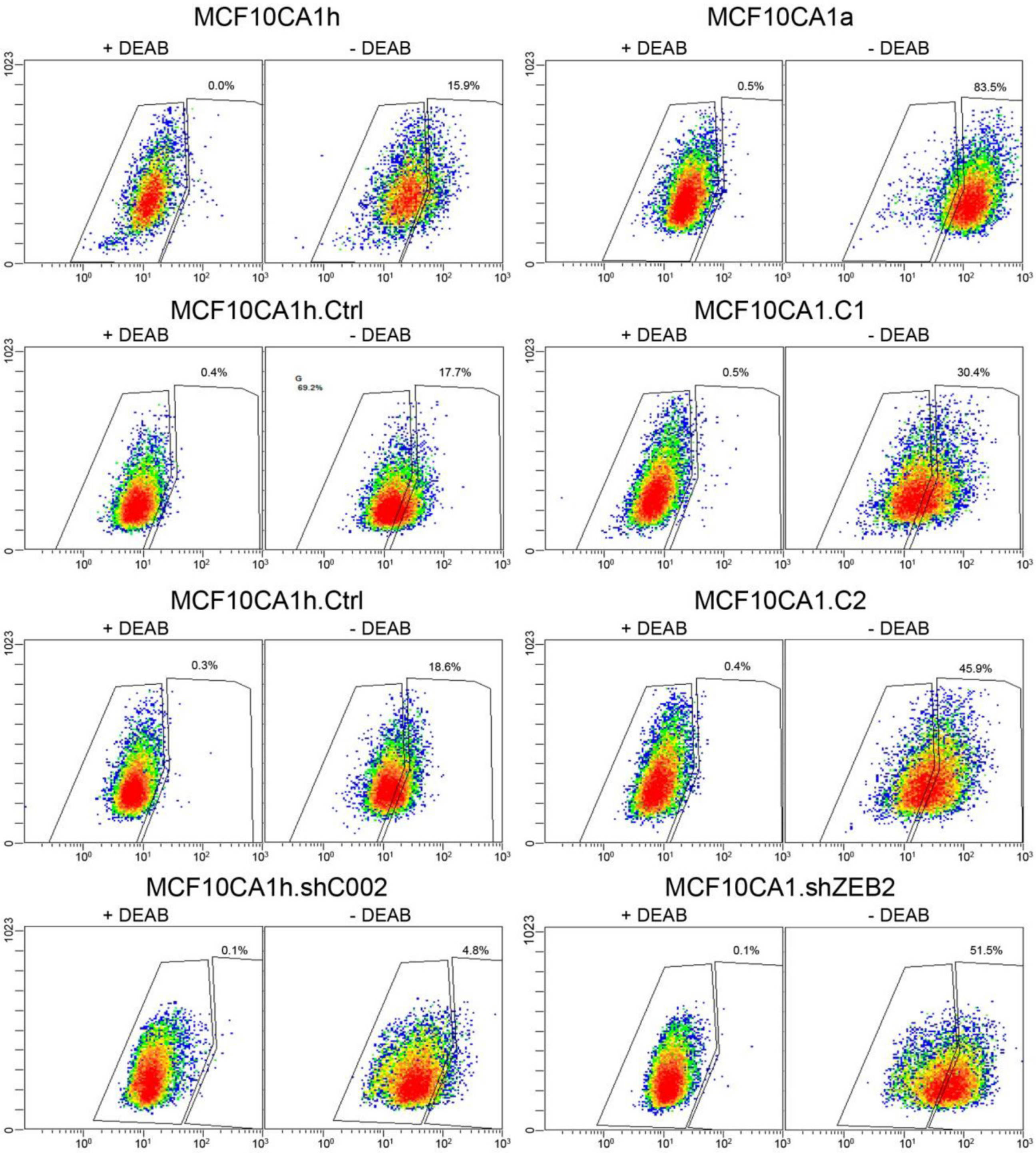

Supplementary Figure 3: Strong induction of aldehyde dehydrogenase (ALDH) activity in MCF10CA1 cells by expression of miR-200s or knockdown of ZEB2. Cells were stably transduced with retroviral vectors for the expression of miR-200-cluster 1 or cluster 2, or with lentiviral vectors for the expression of shZEB2. ALDH activity was determined by flow cytometry with the Aldefluor assay. In order to draw windows of baseline and positive signals, each cell variant was incubated with Aldefluor in the presence of the ALDH inhibitor N,N-diethylaminobenzaldehyde (DEAB).

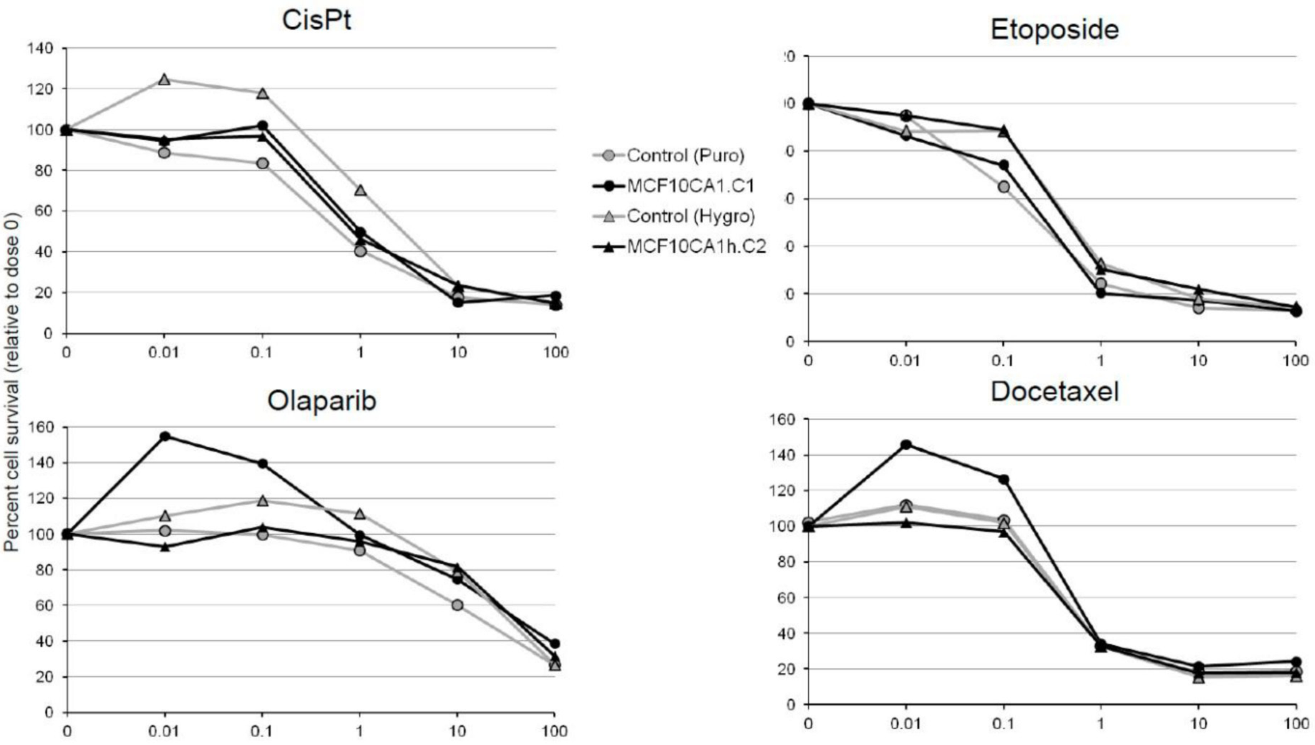

IC50	Puro	C1	Hygro	C2
**cisPt**	0.59720405	0.88857985	4.033593	1.6100005
**Etoposide**	0.30416852	0.3040278	0.52976514	0.61406194
**Olaparib**	61.527077	68.5011578	25.0116725	22.6532711
**Docetaxel**	0.84602233	1.32636575	0.77642979	0.70897229

Supplementary Figure 4: Cluster 1 miR-200's confer variable and moderate resistance of MCF10CA1h cells to cytotoxic drugs. Cells were plated in 96-well plates and subjected for 96 h to varying concentrations of drugs and relative cell numbers determined by the Hoechst 33342 fluorescent assay.

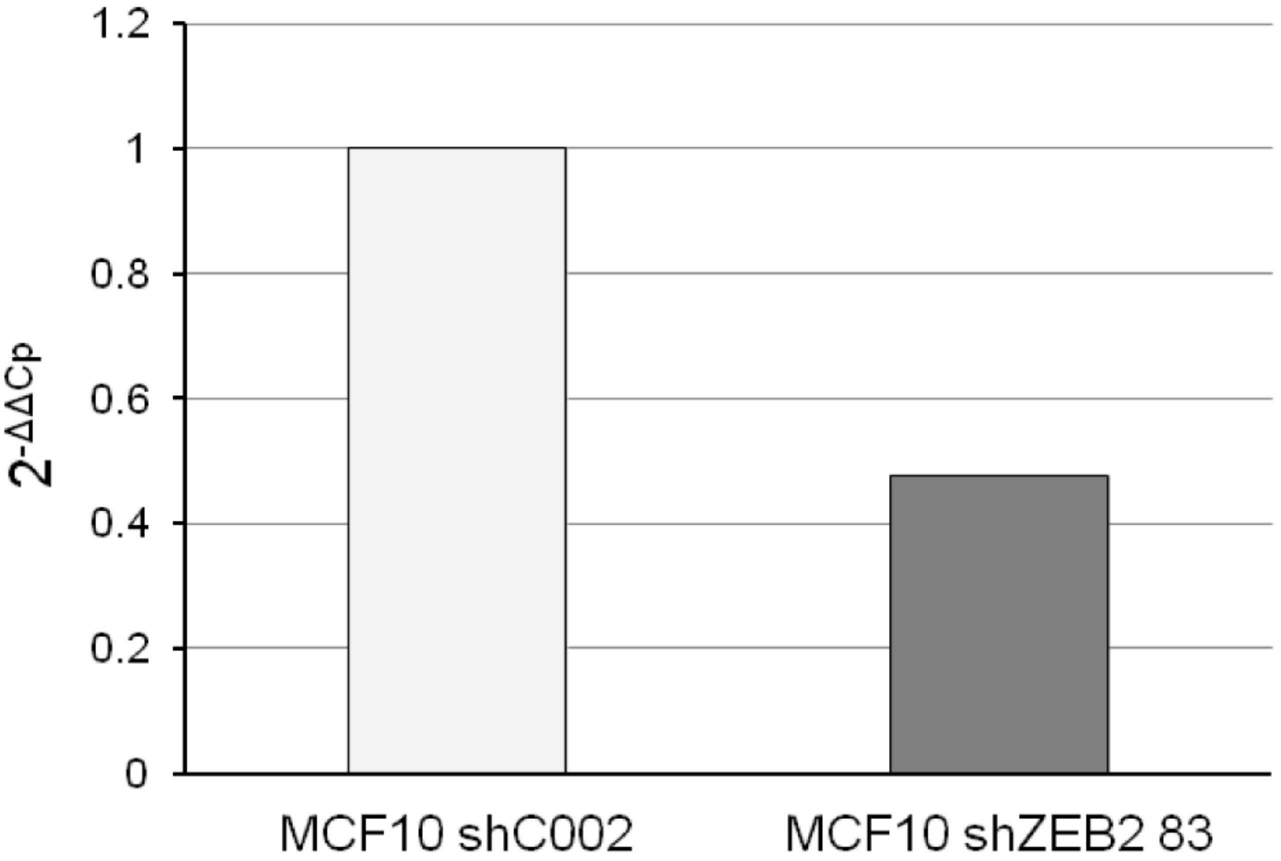

Supplementary Figure 5: Efficient knockdown of ZEB2 in MCF10CA1h cells expressing ZEB2-targeting shRNA. Cells were transduced with lentiviruses expressing control (shC002) or ZEB2-specific shRNAs, selected with puromycin and total RNA purified. Expression levels were determined by qPCR with the UPL system using ZEB2-specific primers and probe combinations. As a reference gene, primer and probe combinations were used to quantify the RPS18 ribosomal protein mRNA. Values in the y axis correspond to $2^{-\Delta\Delta Cp}$.

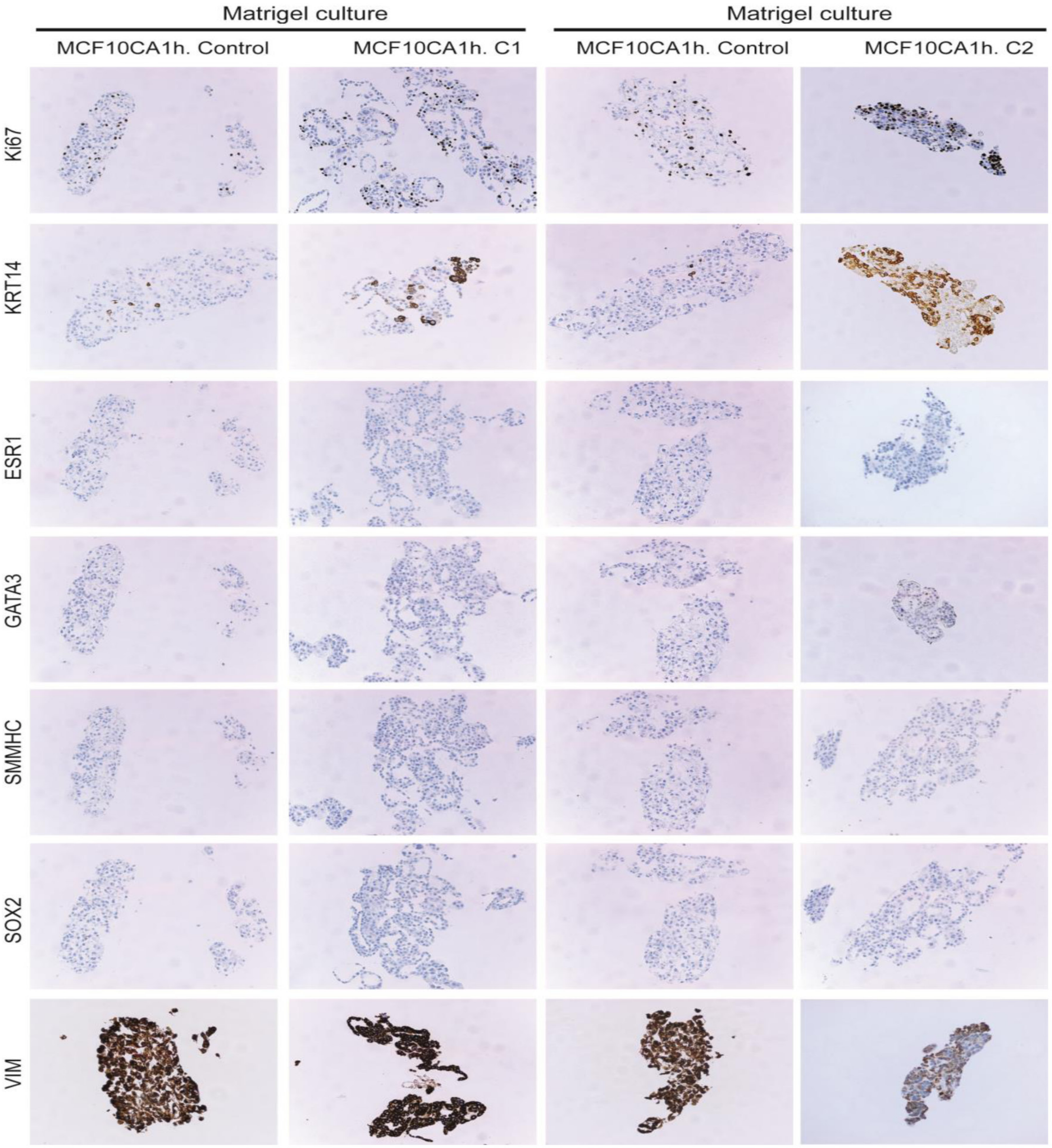

Supplementary Figure 6: miR-200's fail to induce additional markers of luminal (GATA3, ESR1) or myoepithelial terminal differentiation (SMMHC) under differentiating culture conditions. Cells were formalin-fixed and paraffin-embedded and processed for immunostaining and diaminobenzidine-based detection.

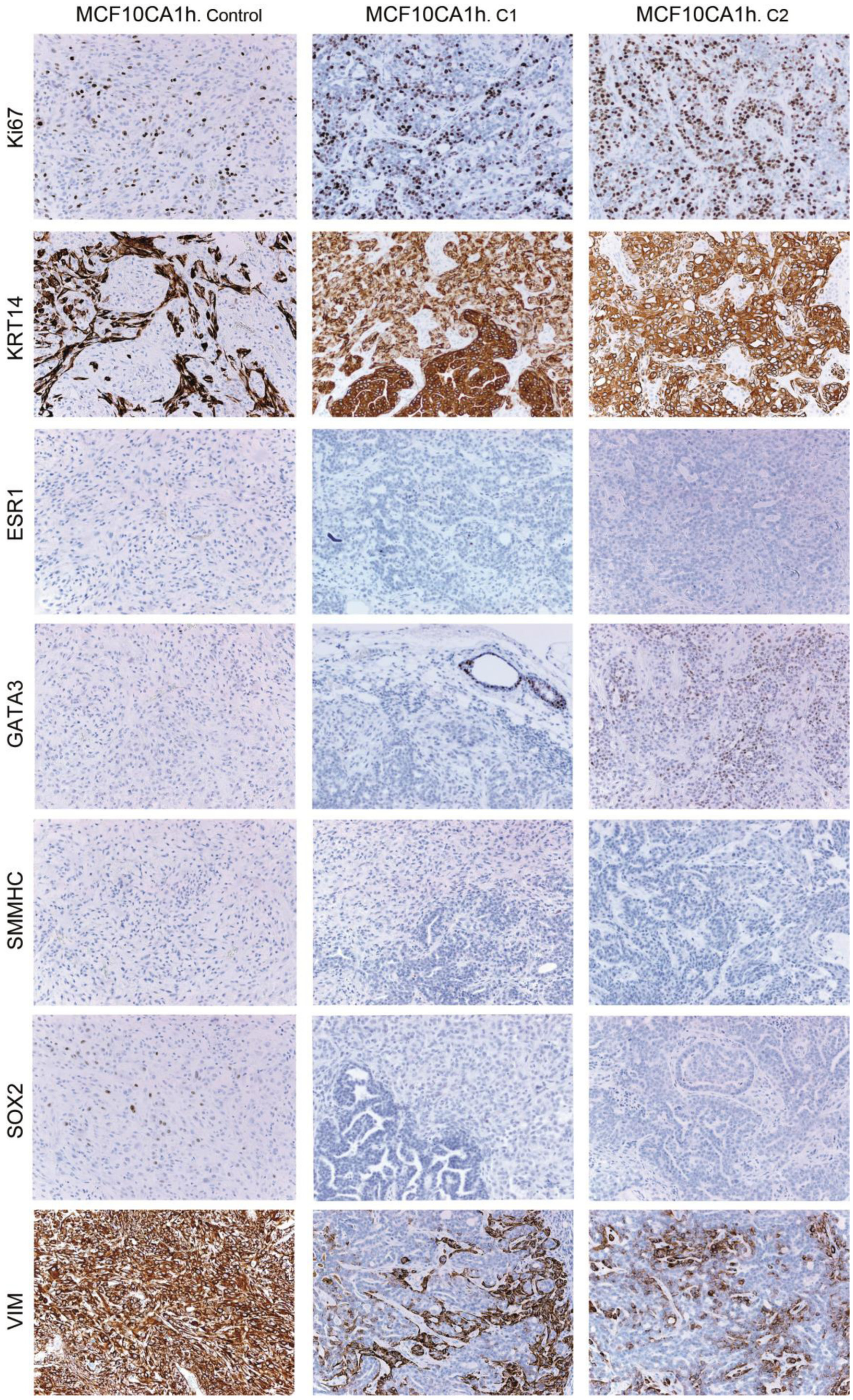

Supplementary Figure 7: miR-200's induce the luminal KRT14 but fail to induce additional markers of luminal (GATA3, ESR1) or myoepithelial terminal differentiation (SMMHC) in orthotopic tumors. In contrast, they strongly downregulate the mesenchymal marker vimentin (VIM). Orthotopic tumors were formalin-fixed and paraffin-embedded and processed for immunostaining and diaminobenzidine-based detection.

For Supplementary Table1 & 2 see in Supplementary Files.

Supplementary Table 3: Evaluation of immunohistochemical staining for the indicated antibodies on metaplastic breast cancer samples.

Case #	E-cadherin E/M (%)	ALDH E/M (%)	KRT8 (Cam5.2) E/M (%)	KRT5 E/M (%)	KRT5 + KRT8/18 E/M (%)	Ki67 E/M (%)	Type
1	10/0	20/60	60/0	10/0	20/0	20/10	carcinosarcoma
2	100/0	60/10	80/20	100/10	100/80		carcinosarcoma
3	100/30	10/50	30/5	0/0	0	10/1	carcinosarcoma
4	60/0	<1/60	5/0	5/0	50/0	10/1	carcinosarcoma
5	90/0	<1/90	5/0	90/0	5/0	2/5	carcinosarcoma (squamous)

E/M (%): percentages of positive cells in the epithelial or mesenchymal components

Supplementary Table 4: **List of antibodies, dilutions and antigen retrieval conditions used for immunostaining of FFPE samples (human and experimental tumors).**

Antibody	Clone	Source	Dilution	Manufacturer	Pretreatment
Cytokeratin 18	H-80	rabbit	1:100	Santa Cruz	High pH
Cytokeratin 5/6	D5/16 B4	mouse	ready-to use	Dako	High pH
Cytokeratin 14	LLOO2	mouse	1:100	Cell Marque	High pH
E-cadherin	NCH-38	mouse	ready-to use	Dako	High pH
Estrogen Receptor α	EP1	rabbit	ready-to use	Dako	High pH
GATA-3	HG3-31	mouse	1:200	Santa Cruz	High pH
Ki-67	MIB-1	mouse	ready-to use	Dako	Low pH
p63	4A4	mouse	ready-to use	Roche	CC1
Smooth Muscle Actin	1A4	mouse	ready-to use	Dako	High pH
Vimentin	V9	mouse	ready-to use	Dako	Low pH
Sox2	D6D9	rabbit	1:30	Cell Signalling	Low pH

CC1: Cell Conditioning Solution (CC1) (Ventana, Roche), a tris based buffer with a slightly basic pH; antigen retrieval at 95º for 30 minutes
High pH: pH 9 Tris/EDTA buffer; EnVision™ FLEX Target Retrieval Solution (Dako Omnis)
Low pH: pH 6.1 citrate buffer; EnVision™ FLEX Target Retrieval Solution (Dako Omnis)

Supplementary Table 5: **Primers and UPL probes used for real-time RT-PCR quantification of mRNAs**

Gene	UPL probe	Forward 5'-3'	Reverse 5'-3'
CDH1	35	CCCGGGACAACGTTTATTAC	GCTGGCTCAAGTCAAAGTCC
EPCAM	3	CCATGTGCTGGTGTGTGAA	TGTGTTTTAGTTCAATGATGATCCA
ZEB1	3	GGGAGGAGCAGTGAAAGAGA	TTTCTTGCCCTTCCTTTCTG
ZEB2	68	AAGCCAGGGACAGATCAGC	GCCACACTCTGTGCATTTGA
KRT8	64	GATGAACCGGAACATCAGC	CATCCTTAATGGCCAGCTCT
KRT18	78	TGATGACACCAATATCACACGA	GGGCTTGTAGGCCTTTTACTTC
KRT5	1	GCAGATCAAGACCCTCAACAAT	CCACTTGGTGTCCAGAACCT
KRT14	18	CCTCCTCCCAGTTCTCCTCT	ATGACCTTGGTGCGGATTT
TP63	10	CGCCATGCCTGTCTACAA	TGACTAGGAGGGGCAATCTG
ACTA2	58	CTGTTCCAGCCATCCTTCAT	TCATGATGCTGTTGTAGGTGGT
SOX2	19	ATGGGTTCGGTGGTCAAGT	GGAGGAAGAGGTAACCACAGG
POU5F1	52	GTGCCTGCCCTTCTAGGAAT	GGCACAAACTCCAGGTTTTCT
KLF4	82	GCCGCTCCATTACCAAGA	TCTTCCCCTCTTTGGCTTG
MYC	34	CACCAGCAGCGACTCTGA	GATCCAGACTCTGACCTTTTGC
NANOG	69	ATGCCTCACACGGAGACTGT	AGGGCTGTCCTGAATAAGCA
BMI1	54	TGTAAAACGTGTATTGTTCGTTACC	CAATATCTTGGAGAGTTTTATCTGACC
FOXA1	1	AGGGCTGGATGGTTGTATTG	ACCGGGACGGAGGAGTAG
ESR1	24	TTACTGACCAACCTGGCAGA	ATCATGGAGGGTCAAATCCA
ELF5	87	AGTTGAGCAGAGCCCTGAGA	GCCTGGAGCAGATCATAGCTT
PTEN	48	GGGGAAGTAAGGACCAGAGAC	TCCAGATGATTCTTTAACAGGTAGC
TSC1	65	CAACCAGAGCCAGGAATTACA	CAGCTCCGCAATCATGTTC
SYNJ1	84	CTGCTTGGTGAAGATGCAGA	GAAGTTCCTCCACTTCAGCACTA
RN18S1	40	GGAGAGGGAGCCTGAGAAAC	TCGGGAGTGGGTAATTTGC

Supplementary Table 6: **Primers used to generate constructs for 3' UTR luciferase assays for TSC1 and SYNJ1**

3' UTR Reporter	Forward 5'-3'	Reverse 5'-3'
SYNJ1	CTCGAGAGCCAAGATTTGCTGTTAGAAAT	GCGGCCGCTTCATATGACAGCACGTTTCAC
TSC1 (F1)	CTCGAGAAAGACTTGGGTGTGGAAGC	GCGGCCGCTGAACTTGCACTCAGACCCT
TSC1 (F2)	CTCGAGTCTGGGTGTGACTGATTCCC	GCGGCCGCACATTGGCCAAACAGCTGAG
TSC1 (F3)	CTCGAGCAGCGCCACATTATCCATCC	GCGGCCGCTCTTTCACTGATGGGACCCC

Supplementary Table 7: Primers used for Chromatin Immunoprecipitation experiments

3' UTR Reporter	Forward 5'-3'	Reverse 5'-3'
SOX2	GGATGGTTGTCTATTAACTTGTTC	AAACACTCTCTTCTCTGCC
KRT5	GTGCAAACGCTTTCTTCC	ACTTGCTGCCAAGAGAT
KRT8	TGGCTCTGGCGGAAT	CCTCTTGTTTACTCCTAGATGAC
KRT18	CAAACTCGCCAGCAC	GGGCAGGAAATGACGTTA
NEUROD1	TTGCGCTCTCTTATGTGG	GCAGAATACAACCGTCAAAGA
ACTB	CTCTAAGGCTGCTCAATGT	GCCAACTTGTCCTTACCC
